# Dimethyl fumarate reprograms cervical cancer cells to enhance antitumor immunity by activating mtDNA-cGAS-STING pathway

**DOI:** 10.1186/s12929-025-01187-x

**Published:** 2025-10-20

**Authors:** Han Jiang, Liting Liu, Shan He, Shen Qu, Yifan Yang, Guijie Kang, Min Wu, Hangyu Liu, Yuwei Zhang, Zixuan Wang, Wenjing Tian, Ying Chen, Liming Wang, Qiangqiang Wang, Ting Ye, Junyan Han, Hui Wang, Yafei Huang

**Affiliations:** 1 Department of Pathogen Biology, School of Basic Medicine, Tongji Medical College, Huazhong University of Science and Technology, Wuhan, China; 2 Department of Gynecologic Oncology, Women’s Hospital, School of Medicine, Zhejiang University, Zhejiang, China; 3 Zhejiang Provincial Key Laboratory of Precision Diagnosis and Therapy for Major Gynecological Diseases, Women’s Hospital, School of Medicine, Zhejiang University, Hangzhou, Zhejiang China; 4 Department of Obstetrics and Gynecology, Tongji Hospital, Tongji Medical College, Huazhong University of Science and Technology, Wuhan, China; 5 Cancer Center, Department of Medical Oncology, Zhejiang Provincial People’s Hospital (Affiliated People’s Hospital), Hangzhou Medical College, Hangzhou, Zhejiang China; 6 Department of Immunology, School of Basic Medicine, Tongji Medical College, Huazhong University of Science and Technology, Wuhan, China; 7 Cancer Center, Union Hospital, Tongji Medical College, Huazhong University of Science and Technology, Wuhan, China; 8 State Key Laboratory for Diagnosis and Treatment of Severe Zoonotic Infectious Diseases, Huazhong University of Science and Technology, Wuhan, China; 9 Key Laboratory of Organ Transplantation, Ministry of Education; NHC Key Laboratory of Organ Transplantation; Key Laboratory of Organ Transplantation, Organ Transplantation Clinical Medical Research Center of Hubei Province, Chinese Academy of Medical Sciences, Beijing, China

**Keywords:** Immunotherapy, CD8^+^ T, CXCL10, CCL5, Dimethyl fumarate, Cervical cancer

## Abstract

**Background:**

Cervical cancer (CC) remains a significant global health challenge for women, especially in advanced stages where effective treatments are limited. Current immunotherapies, including PD-1/PD-L1 blockades and adoptive T cell therapies, show limited response rates and durability. Dimethyl fumarate (DMF), an FDA-approved drug for autoimmune diseases, has demonstrated direct antitumor activity in several cancers. However, its influence on anti-tumor immunity and its function in CC remain poorly understood. This study aims to investigate the therapeutic potential of DMF in CC models and elucidate its underlying mechanisms of action.

**Methods:**

CC cell lines and mouse models were treated with DMF. Transcriptomics profiling of cervical cancer cells following DMF treatment were analyzed by RNA-seq and bioinformatic methods. Mitochondrial DNA (mtDNA) release, and cGAS-STING activation were assessed via qPCR, immunofluorescence, immunoblotting and ELISA. CD8^+^ T cell recruitment was analyzed by flow cytometry. Combinatorial therapies (DMF + anti-PD-1/TILs) were tested in syngeneic or patient-derived xenografts (PDX) models.

**Results:**

DMF treatment induces mitochondrial dysfunction in tumor cells, resulting in the release of mtDNA into the cytosol. The cytosolic mtDNA in turn activates the cGAS-STING-TBK1 pathway and type I interferon response, leading to the secretion of CCL5 and CXCL10, thereby enhancing CD8⁺ T cell infiltration. Additionally, DMF exhibits synergistic effect with PD-1 blockade in murine CC model, and can enhance the therapeutic efficacy of adoptively transferred T cells toward CC in patient-derived xenografts model.

**Conclusion:**

This work elucidated that DMF reprograms CC cells to activate the mtDNA-cGAS-STING pathway, fostering a chemokine-rich microenvironment that recruits CD8^+^ T cells. The synergistic effect of DMF and PD-1 blockade or TIL therapy underscores its potential as an immunostimulatory adjuvant. These findings suggest that DMF holds promise as a novel immunotherapeutic strategy for improving clinical outcomes in CC.

**Graphical Abstract:**

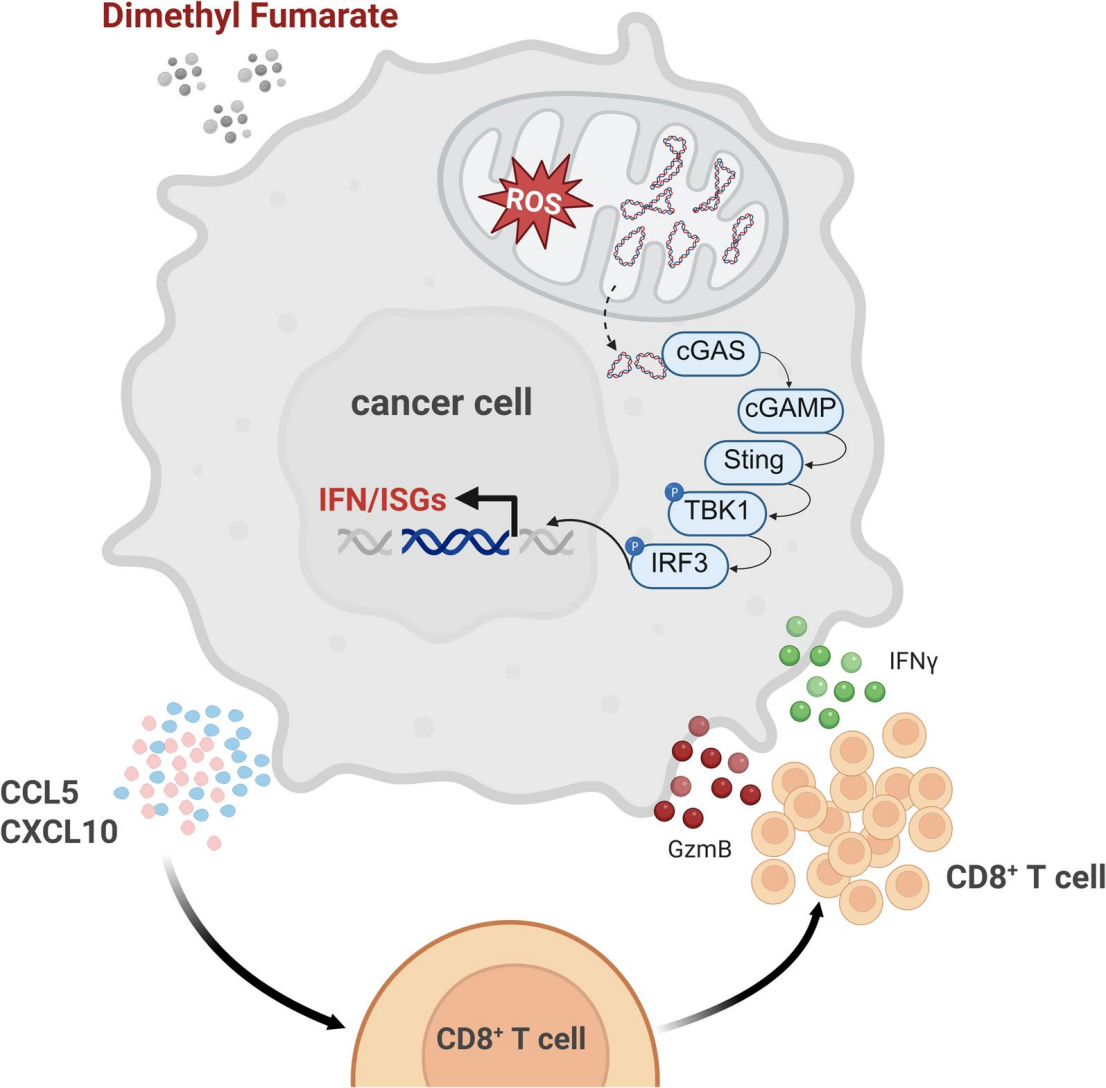

**Supplementary Information:**

The online version contains supplementary material available at 10.1186/s12929-025-01187-x.

## Introduction

Cervical cancer (CC) is one of the most common malignant tumors in women, ranking first in incidence and third in mortality among gynecological tumors worldwide, and also occupying a leading position among female malignancies [1–3]. While early-stage CC is often curable through surgery, radiotherapy, and chemotherapy, effective treatments for late-stage and recurrent metastatic cases are still lacking. As a result, these patients suffer from a poor prognosis, with an overall survival of 7 to 9 months and a 5-year survival rate of 15.5% [4]. Therefore, developing new therapeutic strategies is urgently needed.

Tumor immunotherapy has gained attention in CC treatment due to its promising potential [5]. However, current immunotherapies still fall short of clinical expectations. Approved immune checkpoint blockades (ICBs), such as anti-PD-1/PD-L1 antibodies, show objective response rates of only 14.6%-27.8% in advanced CC patients [6–8], highlighting issues with immunogenicity and durability. Meanwhile, as a promising therapeutic modality by replenishing tumor-specific T cells, T cell receptor-gene engineered T cell (TCR-T) therapy is limited by antigen selection and MHC restriction, benefiting only a small number of patients [9–11]. This obstacle can be circumvented by tumor-infiltrating lymphocyte (TIL) therapy through targeting multiple tumor antigens; however, the efficacy of TIL varies due to patient heterogeneity [12, 13]. Therefore, improving the efficacy of current immunotherapies is of paramount importance for treating CC patients.

Dimethyl fumarate (DMF) is an FDA-approved clinical drug for the treatment of autoimmune diseases such as multiple sclerosis and psoriasis [14, 15]. It is mainly administered orally and is rapidly converted into its primary active metabolite, monomethyl fumarate (MMF), under the action of esterases in the body. Both DMF and MMF exhibit excellent cell permeability (absorption capacity) [16, 17]. Interestingly, while DMF primarily exerts anti-inflammatory effects in autoimmune diseases, it mediates antitumor effects in cancer. In preclinical models, DMF treatment has been shown to cause regression and/or reduced metastasis in various types of tumors, such as melanoma [18, 19], colorectal cancer [20], breast cancer [21], and lung cancer [22]. However, despite extensive research supporting the antitumor potential of DMF, its antitumor mechanism, particularly its impact on antitumor immunity, remains unclear.

Here, we demonstrated that DMF is capable of reprogramming CC cells to promote anti-tumor immunity through activating the mtDNA-cGAS-STING pathway. DMF treatment induces mitochondrial dysfunction in CC cells, leading to the release of mitochondrial DNA (mtDNA) into the cytoplasm, which in turn activates the cGAS-STING pathway and type I interferon response, and subsequently induces the secretion of CCL5 and CXCL10, thereby promoting CD8^+^ T cell recruitment. Importantly, combinatorial therapy with DMF and PD-1 blockade is superior to monotherapies, suggesting a synergistic effect. Finally, DMF is able to potentiate the therapeutic efficacy of adoptively transferred TILs in the human patient-derived xenografts (PDX) model of CC, suggesting its potential in clinical application.

## Materials and methods

### Cell lines

The murine tumor cell line TC-1 and human tumor cell line SiHa were purchased from the American Type Culture collection (ATCC, USA). All cell lines were cultured in DMEM supplemented with 10% fetal bovine serum (FBS) at 37℃ in 5% CO_2_ and routinely tested for mycoplasma. *Sting* KO, *Cxcl9 KO* and *Ccl5*&*Cxcl10* KO TC-1 cells were generated using CRISPR plasmid vectors for the mouse *Sting* gene (VectorBuilder, VB900088-3817jne), *Cxcl9* genes (VectorBuilder, VB900049-4111kbk) and *Ccl5*&*Cxcl10* genes (VectorBuilder, VB240830-1104jfz), respectively. In brief, the CRISPR plasmid vectors were transfected into target cells using lipofectamine (Invitrogen, L3000015). At 48 h post-transfection, puromycin (5 μg/mL) was added to the culture medium to initiate selection, which continued for 5 days to eliminate non-transfected cells and enrich populations stably expressing the resistance gene. Surviving cells were then seeded by limiting dilution in 96-well plates to establish monoclonal colonies.

### Mice

C57BL/6 J and BALB/c nude mice were purchased from the Beijing Vital River Laboratory (Beijing, China). NOD/ShiLtJGpt-*Prkdc*^em26Cd52^*Il2rg*^em26Cd22^/Gpt (NCG) mice were purchased from GemPharmatech (Nanjing, China). All mice were kept under specific-pathogen-free conditions with controlled temperature (21 °C-23 °C), humidity (30–60%) and light cycle (12 h light/dark) at the Animal Care and Use Committee of Tongji Medical College. Female mice (6–8 weeks old) were used in all experiments.

### Antibodies and reagents

For flow cytometry, fixable Viability Stain (564,406) was purchased from BD; anti-mouse Fc Blocker (S17011E), CD45 (30-F11), CD3ε (145-2C11), CD4 (GK1.5), CD8α (53–6.7), IFN-γ (XMG1.2), TNF-α (MP6-XT22), Granzyme B (QA16A02), perforin (S16009A), CD11b (M1/70), F4/80 (BM8), CD206 (C068C2), CD11c (N418), I-A/I-E (M5/114.15.2), CD103 (2E7), NK1.1 (PK136), Foxp3 (MF23), Ly6C (HK1.4), Ly6G (1A8), CD44 (IM7), CD62L (MEL-14), H-2 Kb/H-2Db (28–8-6); anti-human Fc Blocker (422,305), CD45 (2D1), CD3 (SK7), CD4 (RPA-T4), CD8 (RPA-T8), IFN-γ (4SB3), TNF-α (MAb11), were purchased from Biolegend (USA). For immunoblotting, the following antibodies, i.e., anti-IRF3 mAb (A19717, ABclonal, China), anti-Phospho-IRF3 mAb (4947, CST, USA), anti-TBK1 mAb (A3458, ABclonal), anti-Phospho-TBK1 mAb (5483, CST), and anti-β-actin mAb (AC004, ABclonal) were used. The antibodies used for immunofluorescence were anti-DNA mAb (CBL186, Sigma, USA), anti-TOM20 mAb (A19403, ABclonal), anti-8-OHdG mAb (sc-66036, Santa Cruz), AF488-conjugated Goat anti-Rabbit (AS073, ABclonal), AF594-conjugated Goat anti-Mouse (AS054, ABclonal). Additional reagents included monomethyl fumarate (HY-103252, MCE, China) and dimethyl fumarate (HY-17363, MCE).

### Mouse model

For DMF treatment model, DMF was dissolved in dimethyl sulfoxide (DMSO) and solubilized under ultrasonic conditions at 37 °C. The resulting solution was sequentially mixed with 10% DMSO, 40% PEG300, 5% Tween-80 and 45% saline, ensuring thorough homogenization prior to use for oral gavage administration. For in vivo experiments, 1 × 10^5^ TC-1 cells were subcutaneously implanted into C57BL/6 J wild-type (WT) or BALB/c nude mice. DMF treatment (30 mg/kg) was initiated via oral gavage on day 7 post-inoculation and continued daily for 10 consecutive days. For anti-PD-1 combination therapy, anti-PD-1 antibodies (100 μg per mouse) were administered intraperitoneally on day 10, 13, and 16. For CD4/CD8 depleting model, TC-1 tumor-bearing mice were treated with 100 μg of CD4- or CD8-depleting antibodies on day 3, 6, 9, and 12, post-tumor inoculation.

For in vivo transplant tumor model with inoculation of DMF/MMF-treated TC-1, TC-1 cells were treated with 100 μM MMF or 50 μM DMF for 24 h and then subcutaneously implanted into C57BL/6 J WT or BALB/c nude mice. For animal model examining the immunogenicity of DMF/MMF-treated TC-1 cells, mice that failed to develop tumors after subcutaneous implantation of DMF-pretreated TC-1 cells and control mice injected with PBS, were implanted with untreated TC-1 cells into the contralateral side 30 days later.

For PDX model, the patient-derived tumor tissues and paired TILs were established previously [23]. Tumor tissue blocks with a volume of 3–4 mm^3^ were implanted into NCG mice. Treatment started on day 8 of tumor growth with intravenous adoptive transfer of TILs at a dose of 1 × 10⁷ cells per mouse, administered once every seven days, followed by intraperitoneal injection 45,000 IU IL-2 at the same time as TILs, continuing for three consecutive days and then one-day intervals. On day 15 of tumor growth, oral gavage treatment with 30 mg/kg DMF was initiated, administered once daily for 10 consecutive days.

### RNA-seq

TC-1 cells were treated with 50 µM DMF or DMSO for 24 h. RNA was extracted from these samples and sent to BGI (China, Shenzhen) for sequencing. The cDNA library was constructed using the BGISEQ-500 platform. After obtaining clean reads, they were aligned to the reference genome sequence (GCF_000001635.26_GRCm38.p6) using HISATs. Differential gene expression analysis, Gene ontology (GO) enrichment analysis, Gene set enrichment analysis (GSEA) and heat map analysis were performed by the Dr. Tom online system (BGI-Shenzhen, China).

### qRT-PCR

Total RNA was extracted from cells with FastPure Cell/Tissue Total RNA isolation Kit (RC112, Vazyme, China) and reverse transcribed into cDNA by using HiScript II Reverse Transcriptase (R201, Vazyme). cDNA was used as a template and qPCR was performed using ChamQ Universal SYBR qPCR Master Mix kit (Q711, Vazyme) on a Bio-Rad CFX Connect Real-Time PCR System (v.2.0). The primer sequences were described in Supplementary Table 1.

### Quantification of mtDNA in cytoplasm

For the relative quantification of cytosolic mtDNA, the cytosolic fraction was isolated using the Mitochondria/Cytosol Fractionation Kit (ab65320, Abcam). DNA was extracted from whole-cell and cytosolic fractions using the Microsample Genomic DNA Extraction Kit (DP316, TIANGEN, China). qPCR was performed on whole-cell extracts and cytosolic fractions using mtDNA or nucDNA primers (Supplementary Table 2). The Ct values obtained from the DNA of whole-cell extracts were used as a normalization control for the DNA values obtained from cytosolic fractions, enabling effective sample standardization.

### Preparation of tumor single cell suspension

Mouse tumor tissues were minced and digested in 2 ml of DMEM containing 1 mg/ml Collagenase D (11,088,866,001, Roche, Switzerland), 10 μg/ml DNase (abs47047435, Absin, China), and 1% FBS at 37 °C with shaking at 80 rpm for 1 h. After filtration through a 70 μm cell strainer, red blood cells were lysed using red blood cell lysis buffer. The remaining single-cell suspension was used for flow cytometric analysis or magnetic-activated cell separation (MACS).

### Naïve CD8^+^ T cells activation

Murine CD8^+^ T cells were isolated from spleens by positive selection using naive CD8α^+^ T cell Isolation Kit (130–096-543, Miltenyi, Germany). Naïve CD8^+^ T cells were treated with 100 μM MMF or 50 μM DMF, and simultaneously cultured with Dynabeads (11452D, Gibco, USA) in Lymphocyte culture medium (Supplementary Table 3) for 72 h.

### Flow cytometric analysis

For flow cytometry staining of single-cell suspensions from tumor tissues, cells were first stained with a Fixable Viability Staining dye (564,406, BD, USA) at 4 °C for 20 min to identify dead cells and with Fc blocker for 15 min to prevent non-specific staining. To analyze surface markers, cells were incubated for 30 min at 4 °C with a mixture of fluorescein-labeled antibodies. For intracellular cytokine staining, cells were stimulated with Cell Activation Cocktail (with Brefeldin A) (423,304, Biolegend) for 5 h at 37 °C. The cells were collected, fixed and permeabilized after staining of cell surface markers, then stained with the IFN-γ and TNF-α. Flow cytometry was carried out with the FACSVerse (BD).

### Treatment of tumor cells with N-Acetylcysteine

TC-1 or SiHa cells were pretreated with 10 mM N-acetylcysteine (NAC) (HY-B0215, MCE) for 5 h, followed by the addition of 100 μM MMF or 50 μM DMF. Intracellular ROS levels were assessed using the CellROX assay 12 h after drug treatment. Cytoplasmic mitochondrial DNA (mtDNA) levels, excluding mitochondrial fractions, were quantified by qRT-PCR 24 h after treatment.

### ROS staining

Tumor cells were stained with ROS-sensitive probes using either MitoSOX (HY-D1055, MCE) or CellROX (C10444, Invitrogen) reagents. Briefly, stock solutions were diluted in serum-free cell culture medium to prepare 10 μM MitoSOX working solution or 5 μM CellROX working solution. Tumor cells were harvested by trypsinization and washed twice with PBS. A total of 1 × 10⁶ cells were resuspended in 1 mL of MitoSOX or CellROX working solution and incubated at 37 °C for 30 min. Cells were then centrifuged at 400 × g for 5 min at 4 °C, the supernatant was discarded, and the pellet was washed twice with PBS. Finally, cells were resuspended in serum-free medium and analyzed by flow cytometry.

### Transwell assay

TC-1 cells with vector control, *Cxcl9* KO or *Ccl5*&*Cxcl10* KO were treated with 100 μM MMF or 50 μM DMF for 24 h, followed by drug withdrawal. The treated tumor cells were harvested, and equal numbers of cells were reseeded. Supernatants were collected over the following 24 h and placed in the lower chamber of a transwell system (3421, Corning, USA), with activated CD8⁺ T cells added to the upper chamber. The number of migrated CD8⁺ T cells in the lower chamber was counted after 6 h.

### Immunoblotting

Cells were lysed in RIPA supplement with protease inhibitor and phosphorylation inhibitors at 4℃ for 30 min and the protein concentration was detected using the BCA protein quantification kit (P0012, Beyotime, China). Cell lysates were boiled in SDS sample loading buffer, resolved by 10% SDS-PAGE, and transferred onto Immun-Blot PVDF Membrane (1,620,177, Bio-Rad, USA). Membranes were blocked with 5% BSA in TBST (TBS + 0.1% Tween-20) for 1 h at room temperature and incubated with primary antibodies overnight at 4 °C. Membranes were washed five times with TBST, incubated with secondary antibodies for 2 h at room temperature, washed five times with TBST again, and protein bands were visualized by ECL (HY-K1005, MCE). Quantitative analysis of protein expression was performed using ImageJ (v.1.52).

### Measurement of cytokine levels

For cell culture supernatant, tumor cells were treated with 100 μM MMF or 50 μM DMF for 24 h, followed by drug withdrawal. The treated tumor cells were harvested, and equal numbers of cells were reseeded. Supernatants were collected after another 24 h of incubation. For tumor interstitial fluid, the tumor tissue is placed on a 70 µm filter secured at the top of a 15 ml centrifuge tube. The sample was centrifuged at 4 °C, 2,000 × *g* for 20 min, and the filtrate collected from the lower layer was collected. The levels of CXCL10 and CCL5 from supernatant or tumor interstitial fluid were measured using the mouse ELISA kit (88–56,009, Invitrogen, USA and DY466-05, R&D, USA) according to the manufacturer’s instructions.

### Measurement of cGAMP

To measure 2′3'-cGAMP content, cells were treated with 100 μM MMF or 50 μM DMF for 24 h, and were then collected and washed with cold PBS. The levels of 2′3'-cGAMP from cell lysates were measured using the 2′3'-cGAMP ELISA Kit (501,700, Cayman, USA) according to the manufacturer’s instructions. The control was set to 1.

### Immunofluorescence microscopy

Cells grown on glass slides were fixed with 4% paraformaldehyde, then permeabilized with 0.1% Triton X-100. After blocking with 5% BSA for 1 h at room temperature and incubating with primary antibodies overnight at 4 °C, cells were washed with PBS and incubated with fluorophore-conjugated secondary antibodies for 1 h at room temperature and then washed with PBS. Super-resolution imaging was performed using commercialized High Intelligent and Sensitive SIM (HIS-SIM) provided by Guangzhou CSR Biotech Co. Ltd. Images were acquired using a 100 × /1.5 NA oil immersion objective.

### mtDNA depletion

To induce mtDNA depletion in tumor cells, the cells were cultured in DMEM containing 10% FBS, with the addition of 100 ng/mL ethidium bromide (HY-D0021, MCE) or 100 nM 2',3'-dideoxycytidine (HY-17392, MCE). Culture medium was replaced every 2 days for 20 days.

### Culture of PDXO

The establishment of PDX-derived organoid (PDXO) has been described in detail in previous studies [23]. Briefly, PDXO was cultured at 37 °C in PDXO culture medium (Supplementary Table 4). Under these culture conditions, the transcription level of indicated molecules in PDXOs treated with MMF/ DMF for 24 h was measured by qRT-PCR.

### Statistical analysis

Data are presented as mean ± SD. Statistical analyses for in vivo and in vitro assays involving multiple-group comparisons were conducted using Dunnett’s multiple comparisons test (one-way ANOVA), Sidak’s multiple comparisons test (two-way ANOVA), or Tukey’s multiple comparisons test (two-way ANOVA). For single comparisons, unpaired or paired two-tailed Student’s t-tests were applied as appropriate. In cases of non-Gaussian distribution, nonparametric tests were employed, including the Wilcoxon matched-pairs signed-rank test for paired single comparisons, the Mann–Whitney U test for unpaired single comparisons, and the Kruskal–Wallis test for unpaired multiple-group comparisons. All statistical analyses were performed using GraphPad Prism software (v.8). A *P* value of < 0.05 was considered statistically significant throughout the study.

## Results

### DMF enhances anti-CC immunity through CD8^+^ T Cell-mediated mechanisms

**Fig. 1:**
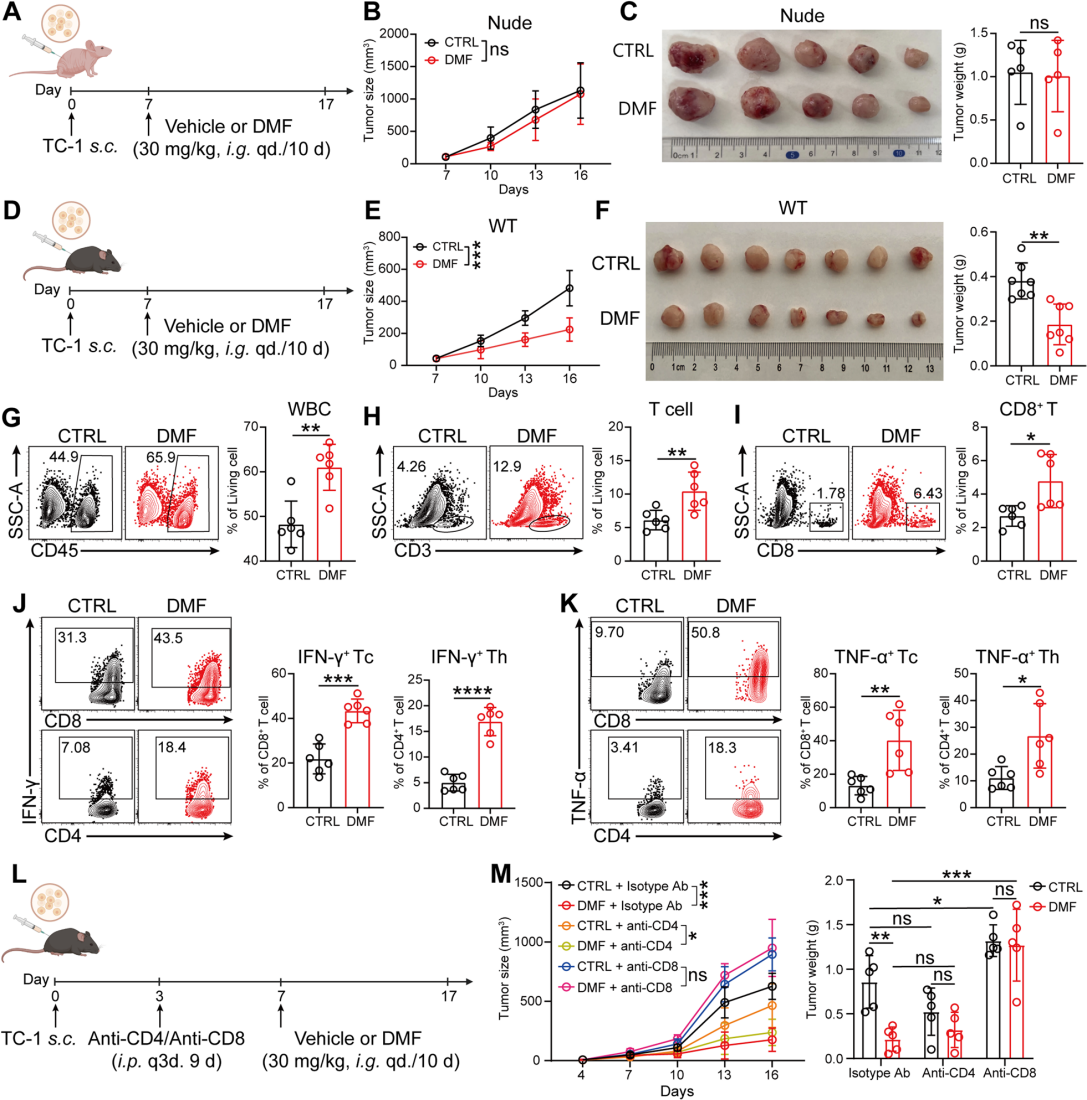
DMF enhances anti-CC immunity through CD8^+^ T Cell-mediated mechanisms. **A**, Schematic of experimental design for **B** and **C**. *i.g*., intragastric administration; *s.c*., subcutaneous. Created with BioRender.com. **B**, Tumor growth curve in TC-1 tumor-bearing nude mice following *i.g*. treatment with DMF. (n = 5 mice). **C**, Image of tumors (left) and weight measurements (right) in nude mice treated with or without 30 mg per kg body weight of DMF at day 17. **D**, Schematic of experimental design for **E** and **F**. Created with BioRender.com. **E**, Tumor growth curve in TC-1 tumor-bearing immunocompetent mice following *i.g*. treatment with DMF. (n = 7 mice). **F**, Image of tumors (left) and weight measurements (right) in immunocompetent mice treated with or without 30 mg per kg body weight of DMF at day 17. **G**–**K**, As in **D**, percentage of infiltrating white blood cells (WBC) (**G**); total T cells (**H**); CD8⁺ T cells (**I**); the proportions of IFN-γ⁺ (**J**) and TNF-α⁺ (**K**) cells among CD8⁺ T cells and CD4⁺ T cells in tumor of immunocompetent mice were analyzed by flow cytometry. (n = 6 mice). **L**, TC-1 tumor-bearing mouse treated with or without DMF and intraperitoneal (*i.p.*) injected with 100 μg of CD4/CD8-depleting antibodies on day 3, 6, 9, and 12 post tumor inoculation. Created with BioRender.com. **M**, Tumor growth curves (left) and tumor weights (right) were measured. (n = 5 mice). Data are the mean ± SD. *P* values were calculated using unpaired two-tailed Student’s *t* test (**B, C, E**–**K**), two-way ANOVA for Tukey’s multiple comparisons test (**M**). *P < 0.05, **P < 0.01, ***P < 0.001, ****P < 0.0001, and ns indicating no significant difference

Previous studies have shown that DMF treatment can result in tumor regression and/or reduced metastasis in several types of cancers [24]. In the quest for validating these findings in CC and exploring the possible mechanisms, we first examined the therapeutic effect of DMF on transplant tumor in immunodeficient (nude) mice generated by TC-1 cell line inoculation (Fig. 1A). Of note, DMF was administered at a clinically equivalent dose (i.e., 30 mg/kg body weight), which has been used in various autoimmune mouse models [25, 26]. We did not note a significant change in tumor growth after DMF treatment in the immunodeficient mice (Fig. 1B, C), although DMF displayed certain tumoricidal effect on CC cells in vitro (Fig. S1A-D). Interestingly, when the same experimental system applied to immunocompetent mice (Fig. 1D), DMF treatment significantly inhibited tumor growth (Fig. 1E, F). These findings thus suggest that although DMF does have anti-CC potential, this effect is dependent on a fully functional immune system.

Immunophenotyping of tumor tissues from immunocompetent mice were performed using flow cytometry (Fig. S1E). Compared to the control group, DMF treatment resulted in a significant increase in the number of infiltrating leukocytes in the tumor tissue (Fig. 1G), with a particularly prominent increase in T cell infiltration (Fig. 1H). Further subset analysis revealed that DMF treatment markedly enhanced CD8⁺ T cell infiltration in tumor tissues (Fig. 1I), whereas the overall proportion of CD4⁺ T cells remained largely unchanged (Fig. S1F). Notably, DMF treatment led to a significant expansion of multiple effector T cell subsets, including IFN-γ⁺CD8⁺ T cells, IFN-γ⁺CD4⁺ T cells (Fig. 1J), TNF-α⁺CD8⁺ T cells, TNF-α⁺CD4⁺ T cells (Fig. 1K), as well as IL-17A⁺CD4⁺ T (Th17) cells (Fig. S1G). These findings suggest DMF may enhance immune-mediated anti-CC responses through the recruitment and activation of inflammatory CD8⁺ and CD4⁺ T cells.

As for other infiltrated immune cells (Fig. S1H), DMF did not significantly alter the proportions of NK cells (Fig. S1I), DCs (Fig. S1J), or conventional DC subsets including cDC1, cDC2, and double-positive DCs (Fig. S1K). It also did not affect macrophage abundance (Fig. S1L) or CD206⁺ macrophage polarization (Fig. S1M). Among immunosuppressive populations (Fig. S2A), DMF significantly increased monocytic myeloid-derived suppressor cells (M-MDSCs) (Fig. S2B) but had no effect on granulocytic MDSCs (G-MDSCs) (Fig. S2B), Tregs (Fig. S2C), or the expression of exhaustion markers PD-1 and TIM-3 on CD8⁺ T cells (Fig. S2D-E).

Next, to confirm the role of T cell subsets in the DMF-mediated antitumor effect, we selectively depleted CD8^+^ T cells and CD4^+^ T cells in the DMF treatment mouse models (Fig. 1L and Fig. S2F). The results showed that depletion of CD8^+^ T cells (Fig. 1M) but not CD4^+^ T cells, significantly reversed the anti-CC effect of DMF, further highlighting the crucial role of CD8^+^ T cells in mediating DMF-induced anti-CC immune responses.

## DMF enhances CD8^+^ T cell-mediated anti-CC immunity by improving the immunogenicity of tumor cells

Given the crucial role of DMF in mediating CD8^+^ T cell-induced anti-CC immune responses, we therefore asked if it’s due to the direct effect of DMF on CD8^+^ T cells. For this purpose, we purified naïve CD8^+^ T cells and treated them with DMF/MMF or medium at the presence of T cell stimuli (Fig. S3A), showing that DMF/MMF treatment significantly inhibited the proliferation of CD8⁺ T cells (Fig. S3B). Moreover, although the cytotoxic proportion of CD8⁺ T cells increased after DMF/MMF treatment, their total number decreased significantly, resulting in a reduction in the absolute number of cytotoxic T cells (Fig. S3C, D). Therefore, the improved infiltration and anti-tumor function of CD8^+^ T cells caused by DMF treatment may result from an indirect mechanism.

We next sought to determine whether DMF modulates immune cells including CD8⁺ T cells in vivo in a tumor cell-independent manner. To this end, tumor-free immunocompetent mice were orally administered DMF at the same dosage used in the tumor model. Flow cytometry analysis of peripheral blood (Fig. S3E) showed no significant changes in the overall proportion of T cells or in any T-cell subsets (Fig. S3 F-N). These results thus indicate that DMF does not directly or broadly alter peripheral immune cell composition. Therefore, the observed immune-stimulating effect of DMF may act through tumor cells.

**Fig. 2:**
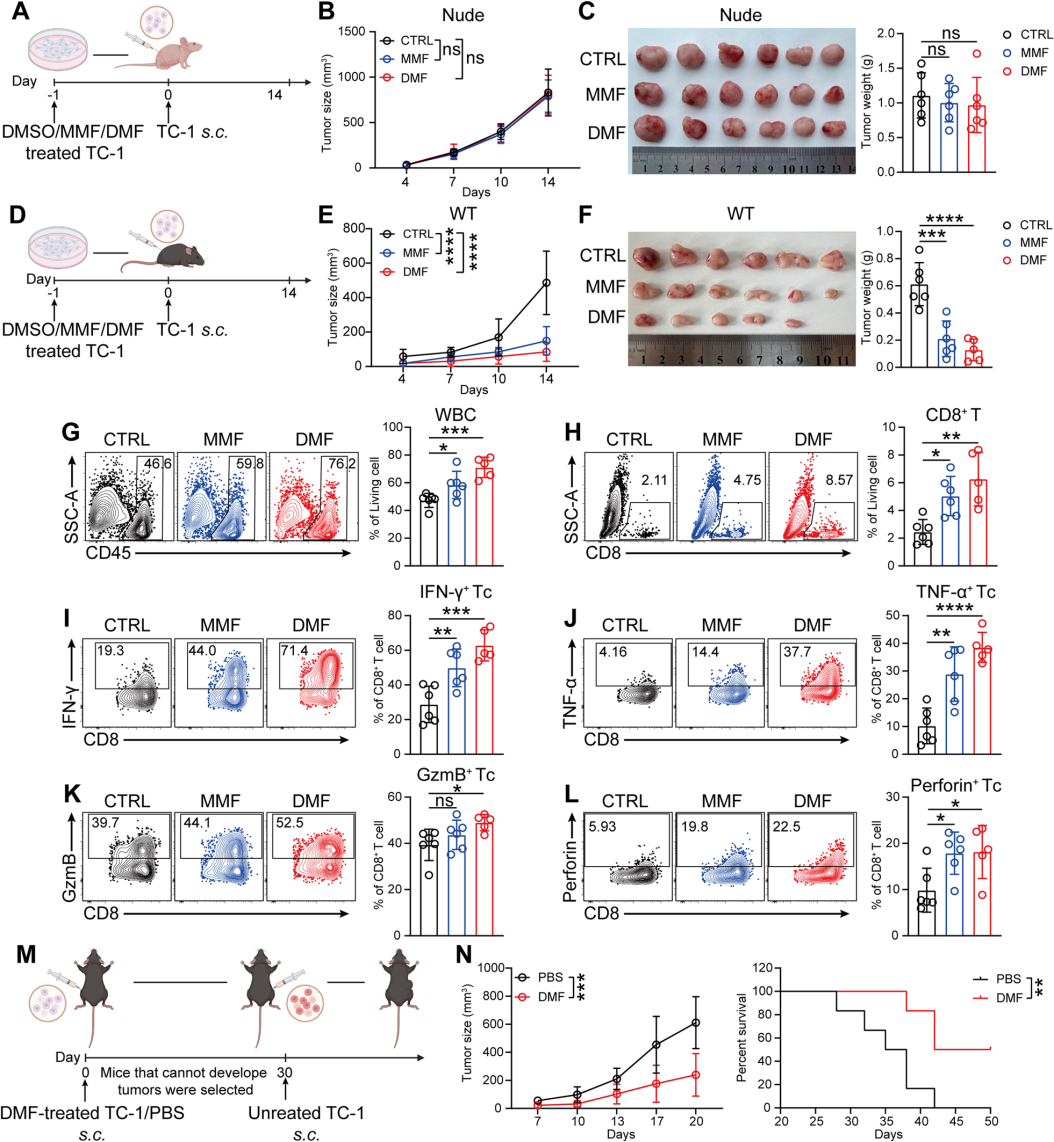
DMF enhances CD8^+^ T cell-mediated anti-CC immunity by improving the immunogenicity of tumor cells. **A**, Schematic of experimental design for **B** and **C**. Created with BioRender.com. **B**, Tumor growth curves. **C**, Image of tumors (left) and weight measurements (right) in immunodeficient mice. (n = 6 mice). **D**, Schematic of experimental design for **E** and **F**. Created with BioRender.com.** E**, Tumor growth curves. **F**, Image of tumors (left) and weight measurements (right) in immunocompetent mice. (n = 6 mice). **G**–**L**, As in **D**, percentage of WBC (**G**); CD8⁺ T cells (**H**); IFN-γ⁺CD8⁺ T cells (**I**); TNF-α⁺CD8⁺ T cells (**J**); GzmB⁺CD8⁺ T cells (**K**) and Perforin⁺CD8⁺ T cells (**L**) in immunocompetent mice were analyzed by flow cytometry. (n = 6 mice). **M**, Schematic of experimental design for **N**. Created with BioRender.com. **N**, Tumor growth curves (left) and mouse survival curves (right) were recorded. (n = 7 mice). Data are the mean ± SD. *P* values were calculated using one-way ANOVA for Dunnett’s multiple comparisons test (**C**, **F**, **G**-**L**), two-way ANOVA for Tukey’s multiple comparisons test (**B**, **E**,** N** left) and log-rank test for survival analysis (**N** right). *P < 0.05, **P < 0.01, ***P < 0.001, ****P < 0.0001, and ns indicating no significant difference

In line with this speculation, previous studies have shown that altering the immunogenicity of tumor cells can influence the function of immune cells within the tumor microenvironment [27, 28]. Therefore, we asked if the antitumor function of DMF is exerted through similar mechanism. To test this, TC-1 cells were treated with DMF or MMF in vitro and were subsequently inoculated into either immunodeficient or immunocompetent mice (Fig. 2A, D). Similar to the in vivo treatment model, DMF/MMF-treated and -untreated TC-1 cells showed no significant growth difference in immunodeficient mice. (Fig. 2B, C). However, in immunocompetent mice, tumor growth was markedly inhibited, with some mice in the DMF-pretreated group failing to develop tumors (Fig. 2E, F). Furthermore, the tumors that were significantly suppressed in growth were accompanied by increased infiltration of CD8⁺ T cells with enhanced effector function (Fig. 2G-L). These results demonstrate that DMF indirectly promotes CD8^+^ T cell-mediated anti-tumor immunity through acting on tumor cells.

Having revealed that DMF enhanced CD8⁺ T cell-mediated antitumor immunity through effects on tumor cells, we next sought to determine whether this effect is accompanied by the induction of immune memory. For this purpose, we first examined T-cell memory subsets in tumor-draining lymph nodes from the DMF-pretreated TC-1 tumor model (Fig. S4A). The distribution of memory subsets within CD4⁺ T cells was unchanged (Fig. S4B). In contrast, DMF treatment significantly increased the proportion of central memory T cells (TCM) within CD8⁺ T cells (Fig. S4C), indicating that DMF promotes the differentiation of antigen-specific CD8⁺ T cells toward a central memory phenotype, which may contribute to durable antitumor immunity.

To confirm the DMF-induced immune memory, we utilized a tumor-reinoculation mouse model. Mice from the DMF-pretreated group that did not develop tumors were reinoculated with tumor cells into the contralateral flank 30 days post-initial inoculation (Fig. 2M). The results showed that, compared to the group injected with PBS on the left side, mice with DMF-pretreated TC-1 cells that failed to form tumors on the left side exhibited significantly slower tumor growth on the right side and had markedly higher survival rates (Fig. 2N). Collectively, these findings suggest that pretreatment of tumor cells with DMF induces a potent and long-lasting anti-tumor immune response through improving the immunogenicity of tumor cells.

## DMF enhances CD8^+^ T cell infiltration through inducing tumor cells to secrete CCL5 and CXCL10

To explore the mechanism by which DMF increases the immunogenicity of tumor cells, we performed RNA-seq analysis on DMF-treated TC-1 cells followed by conducting a customized gene set enrichment analysis. We first focused on the MHC I antigen presentation pathway including genes involved in antigen processing, transport, and surface presentation, which are known essential for activating CD8^+^ T cells. The results showed that the enrichment score for this pathway (*P* = 0.76) did not reach statistical significance (Fig. S5A). Consistently, DMF treatment did not change surface MHC I expression on tumor cells in vivo (Fig. S5B) or alter the expression of MHC I presentation-related genes in the RNA-seq dataset (Fig. S5C). These results indicate that MHC I-mediated antigen presentation on tumor cells is unlikely to contribute substantially to DMF-induced antitumor immunity in cervical cancer.

**Fig. 3:**
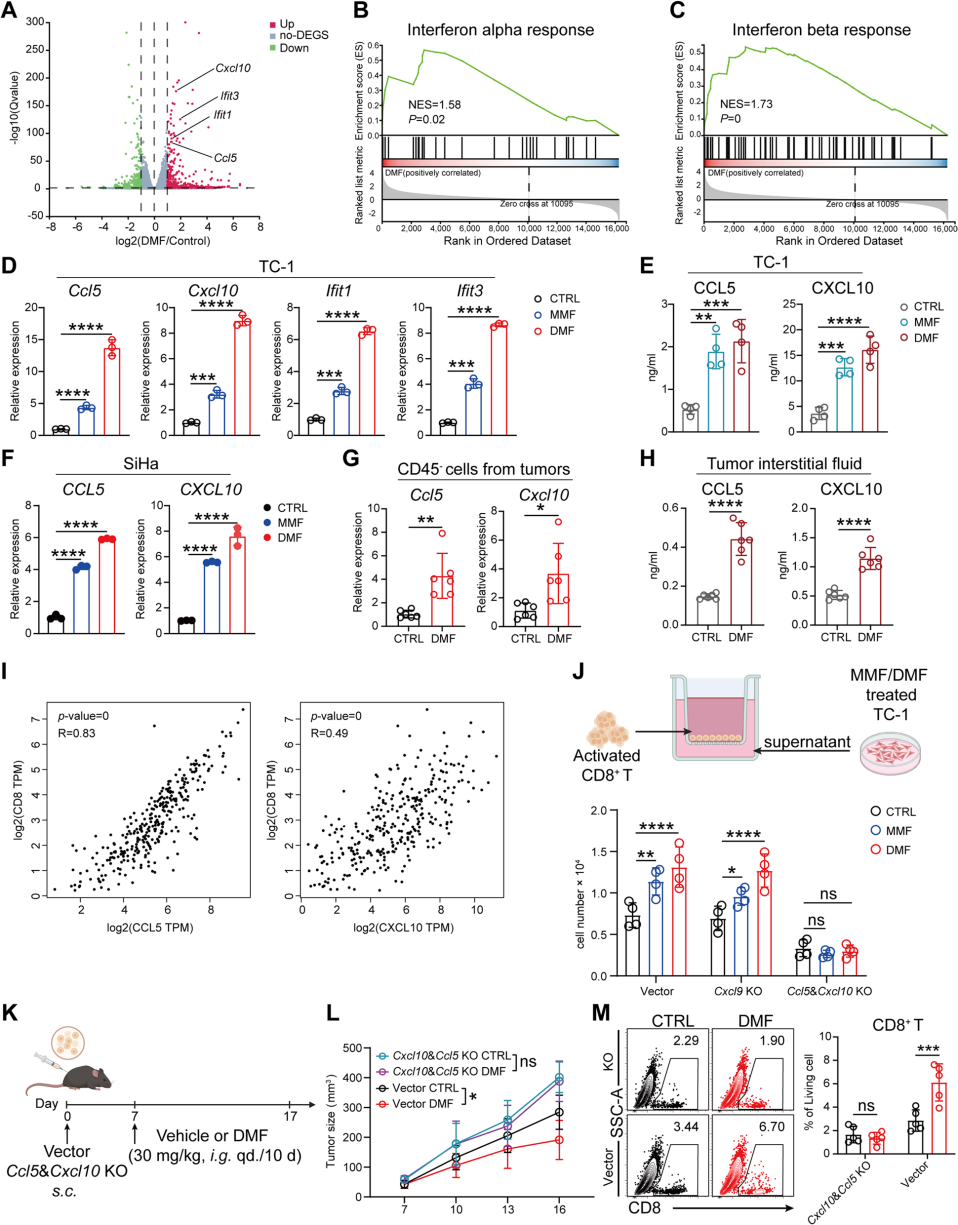
DMF enhances CD8^+^ T cell infiltration through inducing tumor cells to secrete CCL5 and CXCL10. **A**–**C**, RNA-seq results of TC-1 cells treated with or without 50 µM DMF for 24 h. **A**, Volcano plot of differentially expressed genes (DEGs), type I interferon response relative genes were annotated. **B**, GSEA plot showing the interferon alpha response pathway. **C**, GSEA plot showing the interferon beta response pathway. **D**, Transcriptional levels of *Cxcl10*, *Ccl5*, *Ifit1*, and *Ifit3* in TC-1 cells treated with or without 100 μM MMF or 50 μM DMF for 24 h, analyzed by qRT-PCR. **E**, Concentrations of CXCL10 and CCL5 in the supernatant of TC-1 cells after 24 h of treatment with 100 μM MMF or 50 μM DMF, quantified by ELISA. **F**, Transcriptional levels of *CXCL10* and *CCL5* in SiHa cells treated with or without 100 μM MMF or 50 μM DMF for 24 h, analyzed by qRT-PCR. **G** and **H**, After 10 days of treatment with DMF by gavage, tumors were harvested from immunocompetent mice. (as in Fig. 1D). **G**, Transcriptional levels of *Cxcl10* and *Ccl5* in CD45⁻ cells derived from tumor tissue, analyzed by qRT-PCR. (n = 6 mice). **H**, Concentrations of CXCL10 and CCL5 in the tumor interstitial fluid, quantified by ELISA. (n = 6 mice). **I**, Pearson correlation analyses of CD8 mRNA expression with CCL5 and CXCL10 in TCGA-CESC tumor samples. Analysed with http://gepia2.cancer-pku.cn/. **J**, Schematic of experimental design for the Transwell system (left) created with BioRender.com and the number of migrated CD8⁺ T cells in the lower chamber was counted after 6 h. **K**, Schematic of experimental design for **L** and **M**. Created with BioRender.com. **L**, Tumor growth curves. **M**, percentage of CD8⁺ T cells were analyzed by flow cytometry. (n = 5 mice). Data are the mean ± SD. *P* values were calculated using unpaired two-tailed Student’s *t* test (**G** and **H**), one-way ANOVA for Dunnett’s multiple comparisons test (**D**–**F**), two-way ANOVA for Tukey’s multiple comparisons test (**J**,** L** and** M**). *P < 0.05, **P < 0.01, ***P < 0.001, ****P < 0.0001, and ns indicating no significant difference

Next, we compared the gene expression profiles of DMF-treated and untreated TC-1 cells. The volcano plot of differentially expressed genes (DEGs) showed that *Cxcl10*, *Ifit3*, *Ifit1*, and *Ccl5*, which are associated with the Type I interferon pathway [29, 30], were significantly upregulated in DMF-treated tumor cells (Fig. 3A). Consistently, gene set enrichment analysis (GSEA) also indicated an upregulation of the Type I interferon pathway (Fig. 3B, C). Moreover, a comparative analysis of DEGs between the DMF-treated and -untreated groups showed a marked upregulation of multiple type I interferon-responsive genes following DMF treatment (Fig. S5D), which were further confirmed by qRT-PCR (Fig. 3D). Moreover, higher levels of CCL5 and CXCL10 were detected in the supernatant of DMF-treated TC-1 cells, further supporting these findings (Fig. 3E). Additionally, when the human CC cell line SiHa was selected to validate this finding, DMF/MMF treatment again led to upregulation of the CCL5 and CXCL10 genes (Fig. 3F). Importantly, tumor cells extracted from DMF-treated mice (Fig. S5E) exhibited significantly upregulated expression levels of *Ccl5* and *Cxcl10* compared to those from untreated mice (Fig. 3G). Measurement of CCL5 and CXCL10 levels in the tumor interstitial fluid revealed significantly higher concentrations of both chemokines in the DMF-treated group (Fig. 3H). Therefore, our in vitro and in vivo findings together suggest that the heightened type I interferon response, especially increased release of CCL5 and CXCL10 by tumor cells may be responsible for DMF treatment-induced CD8^+^ T cell infiltration.

CCL5 and CXCL10 are well-known for their capacity to recruit CD8^+^ T cells into tissues including tumors [28, 31]. Indeed, CCL5 and CXCL10 were the top two chemokines with increased expression in tumor cells after DMF treatment (Fig. S5F). Retrospective analysis of publicly available gene expression datasets from CC patients revealed that CCL5 and CXCL10 expression correlated positively with CD8⁺ T cell infiltration (Fig. 3I), and that CCL5 expression was also associated with improved prognosis (Fig. S5G). Based on these observations, we hypothesized that CCL5 and CXCL10 mediate DMF-induced CD8⁺ T cell infiltration. Of note, although other chemokines such as CXCL9 can also recruit CD8⁺ T cells [32], our data showed its expression did not significantly change after MMF/DMF treatment (Fig. S5F, H) and was thus used as a negative control in the subsequent experiments.

To confirm the role of CCL5, CXCL10 and CXCL9 in mediating DMF-induced CD8⁺ T cell infiltration, we generated two TC-1 knockout (KO) cell lines: one with simultaneous deletion of *Ccl5* and *Cxcl10* (*Ccl5*&*Cxcl10* KO), and another with deletion of *Cxcl9* (*Cxcl9* KO). Complete loss of the corresponding chemokines was confirmed in culture supernatants or cell lysates (Fig. S5I, J). In transwell migration assays, supernatants from DMF/MMF-treated vector and *Cxcl9* KO cells significantly promoted CD8⁺ T cell migration, whereas supernatants from *Ccl5*&*Cxcl10* KO cells completely lost this ability (Fig. 3J), demonstrating that CCL5 and CXCL10, but not CXCL9, are required for DMF/MMF-induced CD8⁺ T cell recruitment in vitro.

We next examined their role in vivo by implanting *Ccl5* & *Cxcl10* KO or vector TC-1 cells into mice, followed by DMF treatment (Fig. 3K). Consistently, DMF significantly reduced tumor volume and weight and increased CD8⁺ T cell infiltration in control tumors, but these effects were abolished when CCL5 and CXCL10 were absent (Fig. 3L, M). Together, these results establish CCL5 and CXCL10 as essential mediators of DMF-induced CD8⁺ T cell recruitment and antitumor activity in cervical cancer.

## cGAS-STING-TBK1 pathway mediates the DMF-induced anti-tumor immune response

**Fig. 4:**
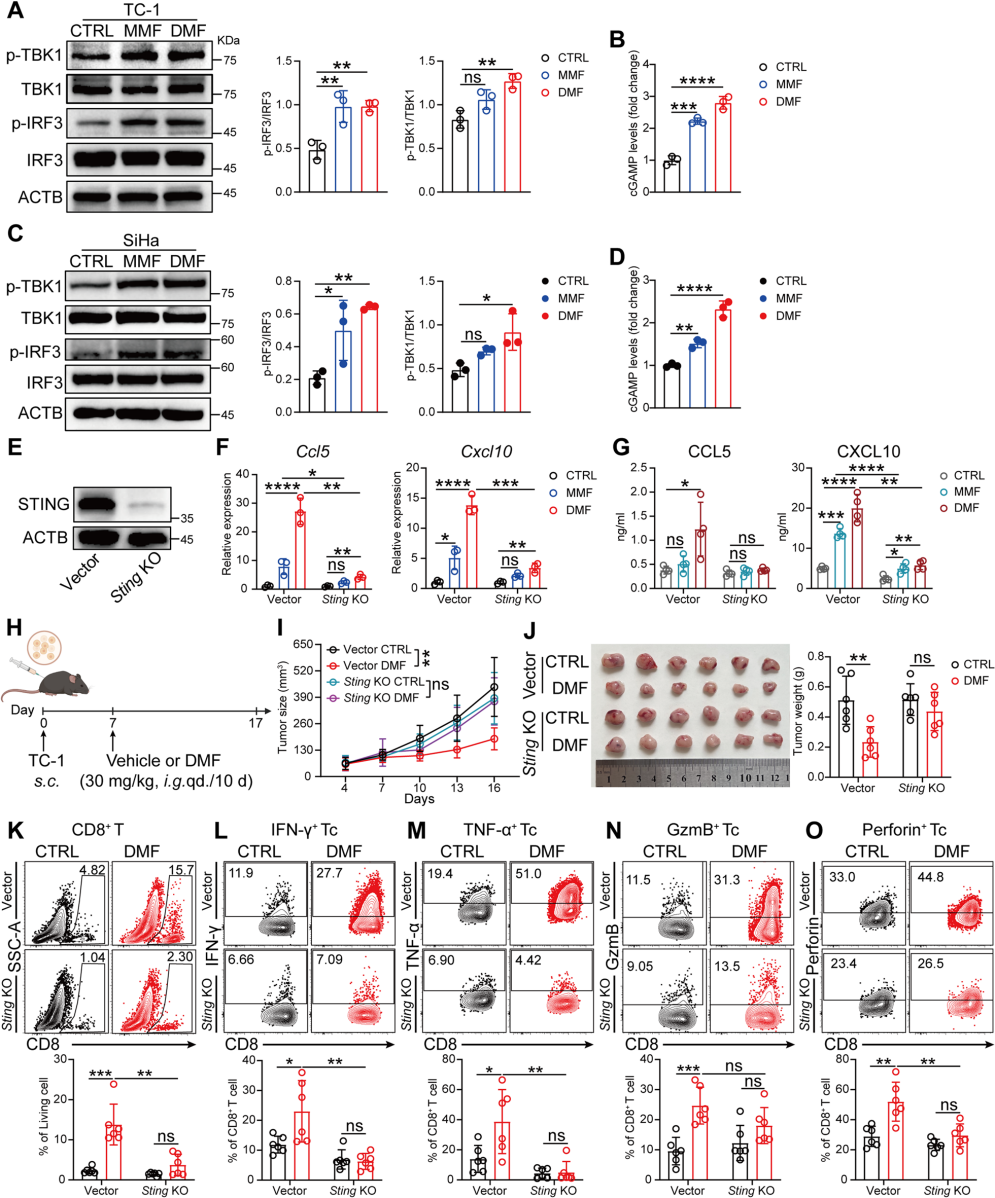
cGAS-STING-TBK1 pathway mediates the DMF-induced anti-tumor immune response. **A** and** B**, Immunoblots of IRF3, p-IRF3, TBK1, p-TBK1 (**A**), and relative abundance of cGAMP (**B**) in TC-1 cells treated with 100 μM MMF or 50 μM DMF for 24 h. **C** and** D**, Immunoblots of IRF3, p-IRF3, TBK1, p-TBK1(**C**), and relative abundance of cGAMP (**D**) in SiHa cells treated with 100 μM MMF or 50 μM DMF for 24 h. **E**, Immunoblots of STING in TC-1 cells, comparing Vector and *Sting* knockout (KO) cells. **F** and **G**, *CCL5* and *CXCL10* were quantified by qRT-PCR (**F**) and ELISA (**G**) respectively, in vector or *Sting* KO TC-1 cells treated with 100 μM MMF or 50 μM DMF for 24 h. **H**–**O**, As in Fig. 1A, a total of 1 × 10^5^ vector or *Sting*^−/−^ TC-1 cells were subcutaneously implanted into mice. **H**, Schematic of experimental design created with BioRender.com. **I**, Tumor growth curves. **J**, Image of tumors (left) and weight measurements (right) at day 17. **K–O**, Percentage of infiltrating CD8⁺ T cell populations, including total CD8⁺ T cells (**K**), IFN-γ⁺CD8⁺ T cells (**L**), TNF-α⁺CD8⁺ T cells (**M**), GzmB⁺CD8⁺ T cells (**N**), and Perforin⁺CD8⁺ T cells (**O**) in tumor were analyzed by flow cytometry. (n = 6 mice). Data are presented as the mean ± SD. *P* values were calculated using one-way ANOVA for Dunnett’s multiple comparisons test (**A**-**D**), two-way ANOVA for Tukey’s multiple comparisons test (**F** and **G, I-O**). *P < 0.05, **P < 0.01, ***P < 0.001, ****P < 0.0001, and ns indicating no significant difference

cGAS-STING-TBK1 pathway has been reported to be pivotal in inducing type I interferon response in the fight against infectious agents and tumor [33]. Therefore, we next explored whether the DMF/MMF-induced anti-CC immune response is regulated by this pathway. Indeed, TC-1 cells exhibited significantly increased expression of p-TBK1 and p-IRF3 (Fig. 4A), along with elevated intracellular cGAMP levels (Fig. 4B) after DMF/MMF treatment. Next, we repeated the experiment in the human-derived SiHa CC cell line and obtained similar results (Fig. 4C, D), indicating that the capacity of DMF/MMF to activate type I interferon response is conserved across CC cells from different species.

Since type I interferon response can be induced through pathways independent of STING (e.g., TLRs), we therefore tested if STING is required for DMF/MMF to activate type I interferon response by generating a *Sting* KO TC-1 cell line (Fig. 4E). The results showed that in *Sting*-deficient TC-1 cells, the upregulation of type I interferon-related genes after DMF/MMF-treatment was significantly reduced compared to that in vector cells (Fig. 4F). Similarly, the levels of CCL5 and CXCL10 in the culture supernatants were also decreased (Fig. 4G). Subsequently, we implanted *Sting* KO TC-1 cells into mice and treated the tumor-bearing mice with DMF (Fig. 4H). In contrast to that observed in WT group, DMF treatment did not significantly affect tumor size in *Sting* KO group (Fig. 4I, J). Moreover, the proportion and function of CD8^+^ T cells infiltrating the tumor tissues remained unchanged (Fig. 4K–O). Together, these results indicate that the cGAS-STING-TBK1 pathway plays a crucial role in DMF-induced antitumor immune responses.

## DMF/MMF treatment induces tumor cells to release mtDNA into the cytoplasm

**Fig. 5:**
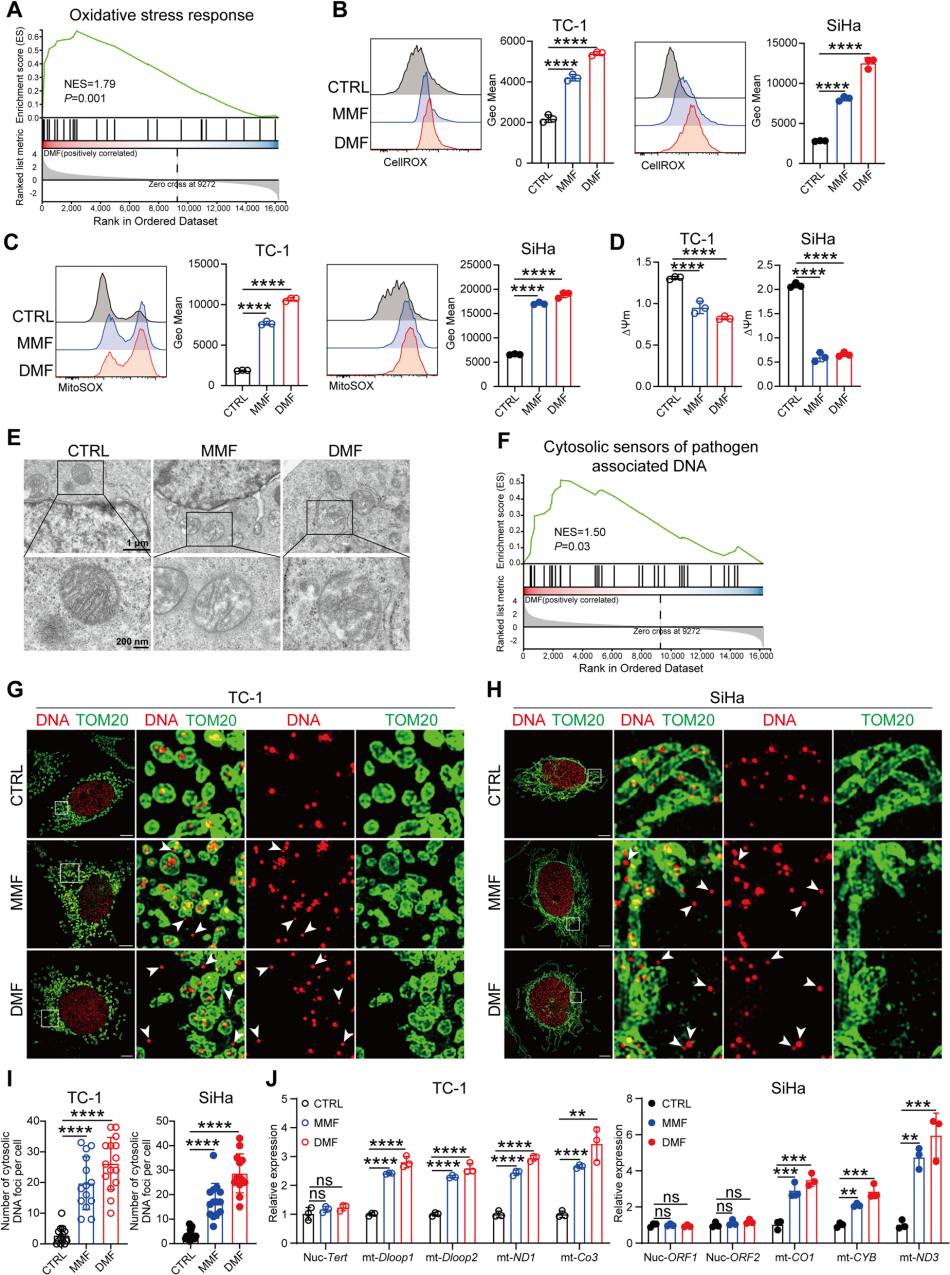
DMF/MMF treatment induces tumor cells to release mtDNA into the cytoplasm. **A**, GSEA plot of the oxidative stress response in TC-1 cells treated with or without 50 μM DMF. **B**–**D**, CellROX levels (**B**), MitoSOX levels (**C**) and mitochondrial membrane potential (**D**) were measured in TC-1 and SiHa cells treated with 100 μM MMF or 50 μM DMF for 12 h. **E**, Electron microscopy of TC-1 cells treated with 100 μM MMF or 50 μM DMF for 24 h. **F**, GSEA plot of the Cytosolic sensors of pathogen associated DNA pathway in TC-1 cells treated with or without 50 μM DMF. **G–H**, Immunofluorescence image of tumor cells treated with 100 μM MMF or 50 μM DMF for 24 h, (**G**) showing TC-1 cells, (**H**) showing SiHa cells. DNA, red; TOM20, green; scale bar = 5 μm. **I**, Quantitative analysis of extramitochondrial DNA in the cytosol in Fig. **G** and **H**. (n = 13–15 cells). **J**, Relative expressions of nuclear (nuc) and mitochondrial (mt) DNA outside the mitochondria in the cytoplasm was detected by qRT-PCR. Data are presented as the mean ± SD. *P* values were calculated using one-way ANOVA for Dunnett’s multiple comparisons test (**B**–**D**,** I**), two-way ANOVA for Tukey’s multiple comparisons test (**J**). *P < 0.05, **P < 0.01, ***P < 0.001, ****P < 0.0001, and ns indicating no significant difference

We went on to investigate the potential mechanisms underlying the upregulation of the cGAS-STING pathway in tumor cells following DMF/MMF treatment. GSEA analysis revealed that genes related to oxidative stress response were significantly enriched in DMF-treated TC-1 cells compared to the control group (Fig. 5A). Further experiments showed that DMF/MMF treatment significantly increased intracellular ROS levels and mitochondrial ROS levels (Fig. 5B, C), while reducing the mitochondrial membrane potential (Fig. 5D), indicating potential mitochondrial damage. Electron microscopy analysis further confirmed mitochondrial cristae disorganization in tumor cells following DMF/MMF treatment (Fig. 5E).

Additionally, GO enrichment analysis indicated a significant increase in cellular response to DNA damage stimulus pathway (Fig. S6A), as well as a notable enrichment in the cytosolic sensors of pathogen associated DNA pathway in the DMF-treated cells (Fig. 5F). Based on these observations, we hypothesize that DMF/MMF-treatment may activate the cGAS-STING pathway by increasing the cytoplasmic DNA content. In line with this hypothesis, the cytoplasmic DNA content was significantly elevated after DMF/MMF treatment in both TC-1and SiHa cell lines, as shown by immunofluorescence staining (Fig. 5G-I). To further verify the source of cytoplasmic DNA (i.e., nuclear or mitochondria) after DMF/MMF treatment, we excluded mitochondria and extracted the DNA from the cytoplasm for qPCR analysis. The results showed that the cytoplasmic DNA in both TC-1 and SiHa cells treated with DMF/MMF is primarily mtDNA rather than nuclear DNA (nucDNA) (Fig. 5J). These results collectively suggest that DMF/MMF treatment induces mitochondrial damage, leading to an increase in cytoplasmic mtDNA content.

DMF treatment increased ROS levels, reduced mitochondrial membrane potential (MMP), and activated DNA damage response pathways, suggesting that mitochondrial ROS production may drive mtDNA release. Indeed, inhibition of ROS generation with N-acetylcysteine (NAC) abolished the DMF/MMF-induced ROS increase (Fig. S6B) and prevented mtDNA release into the extracellular space (Fig. S6C). To determine whether this process was associated with oxidative DNA damage, we measured 8-OHdG levels and found a marked increase following MMF/DMF treatment by flow cytometry (Fig. S6D). Moreover, immunofluorescence revealed that oxidative DNA damage occurred predominantly in the cytoplasm rather than in the nucleus (Fig. S6E, F), indicating that DMF treatment primarily induces oxidative damage to mtDNA.

## Cytoplasmic mtDNA is required for DMF/MMF-induced activation of the cGAS-STING pathway and antitumor response.

**Fig. 6:**
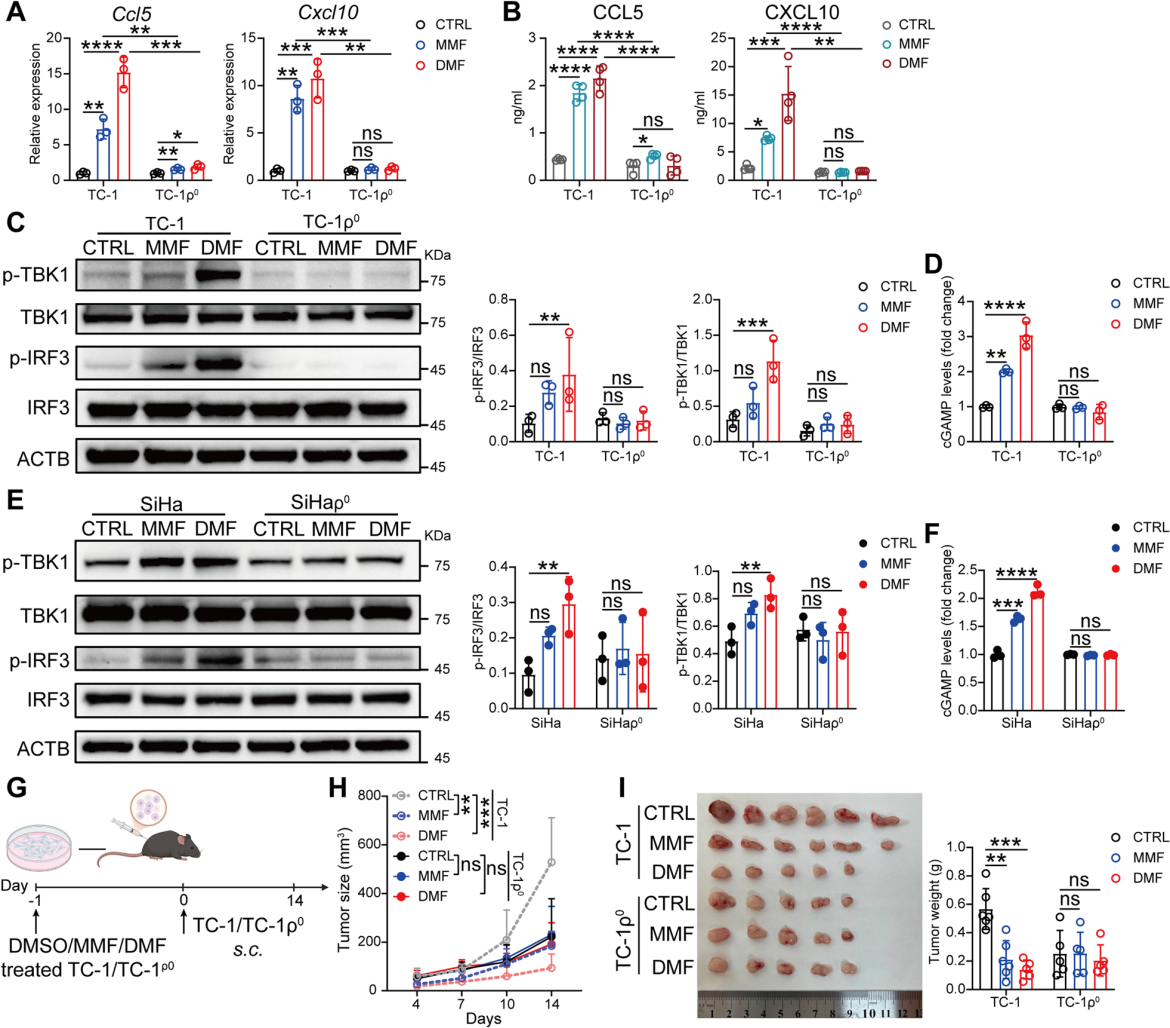
Cytoplasmic mtDNA is required for DMF/MMF-induced activation of the cGAS-STING pathway and antitumor response. **A** and **B**, *CCL5* and *CXCL10* were quantified by qRT-PCR (**A**) and ELISA (**B**) respectively, in TC-1 and TC-1ρ^0^ cells treated with 100 μM MMF or 50 μM DMF for 24 h. **C** and **D**, Immunoblots of IRF3, p-IRF3, TBK1, p-TBK1 (**C**) and relative abundance of cGAMP levels (**D**) in TC-1 and TC-1ρ^0^ cells treated with 100 μM MMF or 50 μM DMF for 24 h. **E** and **F**, Immunoblots of IRF3, p-IRF3, TBK1, p-TBK1 (**E**) and relative abundance of cGAMP levels (**F**) in SiHa and SiHaρ^0^ cells treated with 100 μM MMF or 50 μM DMF for 24 h.** G**, Schematic of experimental design for **H** and **I**. Created with BioRender.com. **H**, Tumor growth curves. **I**, Image of tumors (left) and weight measurements (right) at day 15. (n = 5–6). Data are presented as the mean ± SD. *P* values were calculated using two-way ANOVA for Tukey’s multiple comparisons test (**A-F**,** H** and** I**). *P < 0.05, **P < 0.01, ***P < 0.001, ****P < 0.0001, and ns indicating no significant difference

We next investigated whether the release of mitochondrial DNA into the cytoplasm contributes to the activation of cGAS-STING pathway and the subsequent type I interferon response in DMF/MMF-treated cells by using an established protocol to deplete mtDNA. mtDNA loss was achieved by culturing cells in the presence of either low concentrations of ethidium bromide (EthBr) [34] or 2′,3′-dideoxycytidine (DDC) [35], generating ρ⁰ cells, a derivative of cells that lacks the mitochondrial genome but retains nuclear DNA (Fig. S7A). As expected, depletion of mtDNA significantly reduced the upregulation of type I interferon response-related genes induced by DMF/MMF treatment (Fig. 6A and Fig. S7B, C), and also decreased the secretion levels of CCL5 and CXCL10 (Fig. 6B). Additionally, mtDNA depletion in both TC-1 and SiHa cells suppressed DMF/MMF-induced activation of the cGAS/STING pathway (Fig. 6C–F). Importantly, when mtDNA-depleted TC-1 cells (TC-1ρ^0^) were implanted subcutaneously into mice (Fig. 6G), the inhibited growth of tumors pre-treated with DMF observed in the TC-1 group was no longer noted (Fig. 6H, I). These findings indicate that the release of mitochondrial DNA into the cytoplasm is required for DMF-induced activation of the cGAS-STING pathway and the subsequent anti-CC immune response.

## DMF improve the therapeutic efficacy of immunotherapy with PD-1 blockade and TILs

**Fig. 7:**
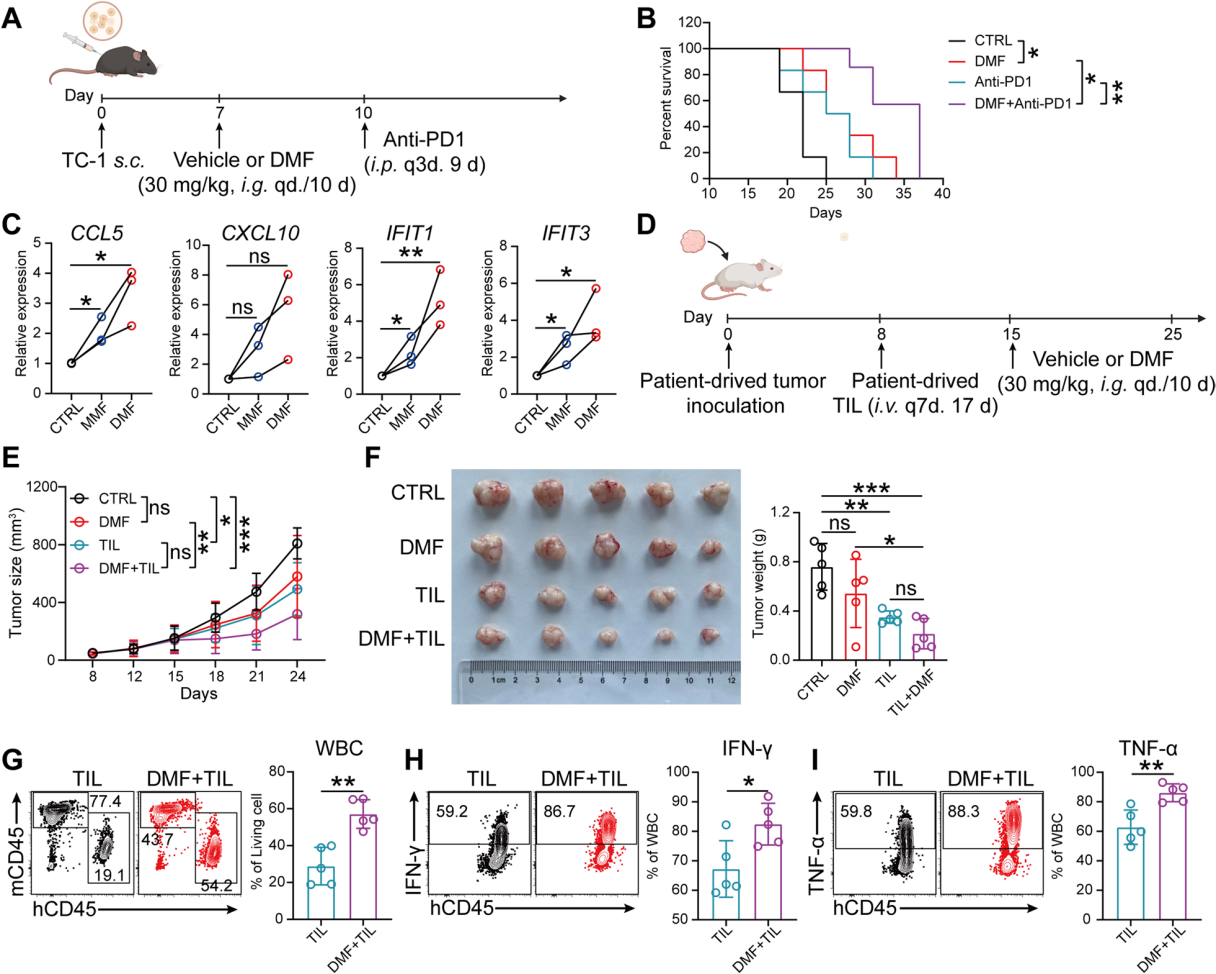
DMF improves the therapeutic efficacy of immunotherapy with PD-1 blockade and TILs. **A,** TC-1 tumor bearing mouse gavaged with or without DMF and treated with 100 μg of anti-PD1 antibodies on day 10, 13 and 16 post tumor inoculation. (n = 6 mice). Created with BioRender.com. **B**, Mouse survival curves were recorded. **C**, Transcriptional levels of *CXCL10*, *CCL*5, *IFIT1*, and *IFIT3* in PDXOs from cervical cancer patients treated with 100 μM MMF or 50 μM DMF, analyzed by qRT-PCR. (n = 3 patients). **D**, Schematic of experimental design for **E**–**F,** *i.v.* intravenous injections. *i.p.* intraperitoneal injections. Created with BioRender.com. **E**, Tumor growth curve. **F**. Image of tumors (left) and weight measurements (right) at day 25. The proportion of patient-derived WBCs (**G**), IFN-γ⁺ WBC cells (**H**) and TNF-α⁺ WBC cells (**I**) in PDX were analyzed. (n = 5 mice). Data are presented as the mean ± SD. *P* values were calculated using unpaired two-tailed Student’s *t* test (**G**–**I**), one-way ANOVA for Dunnett’s multiple comparisons test (**C**), two-way ANOVA for Tukey’s multiple comparisons test (**E** and **F**), log-rank test for survival analysis, and corrected by Bonferroni methods (**B**). *P < 0.05, **P < 0.01, ***P < 0.001, ****P < 0.0001, and ns indicating no significant difference

Although ICB therapy has been approved in CC, patients’ response to this therapy remains relatively low, which is partially attributable to the poor infiltration of ICB-reinvigorated CD8⁺ T cells [6, 36, 37]. Given the capability of DMF treatment in enhancing the infiltration of CD8⁺ T cells, we therefore reasoned that DMF might be able to improve the efficacy of PD-1 blockade by promoting immune infiltration. Indeed, in the TC-1 transplant tumor model, monotherapy with anti-PD-1 antibody did not show significant efficacy, while DMF treatment alone already resulted in markedly prolonged mouse survival. Importantly, combinatorial therapy with DMF and anti-PD-1 antibody were superior to both monotherapies (Fig. 7A, B), indicating the synergistic effect of these two therapeutic regimens.

We next asked if activating cGAS-STING pathway represents a feasible approach for treating human CC. To examine this issue, we first performed association analysis using the public gene database of CC patients, finding that high expression of *STING* is positively correlated with the prognosis of CC (Fig. S7D), suggestive of activating cGAS-STING pathway as a promising therapeutic strategy in treating human CC. To validate this premise, we established PDX tumors from three CC patients and generated corresponding organoid samples (PDXO, Fig. S7E) followed by testing the therapeutic efficacy of DMF toward these human-derived tumors. In line with that noted in murine TC-1 cells (Fig. 3D), PDXO exhibited markedly increased expression of *CCL5*, *CXCL10*, *IFIT1* and *IFIT3* after DMF/MMF treatment (Fig. 7C).

Next, the in vivo evaluation of DMF/MMF’s capacity to induce anti-CC immune response against human tumors and hence tumor regression was performed in a PDX mouse model. As expected, daily treatment with DMF alone starting from day 15 after NCG mice were subcutaneously implanted with PDX tumor failed to inhibit PDX growth given the immunodeficiency status of NCG mice. Therefore, tumor infiltrating lymphocytes (TIL) derived from the same patient where PDX was derived were next prepared (Fig. S7F) and infused at day 8, 15 and 22 post tumor inoculation into mice with or without DMF treatment (Fig. 7D). The results showed that the combination of DMF and TIL effectively inhibited tumor growth, with the therapeutic effect being superior to that of TIL alone (Fig. 7E, F). Flow cytometric analysis of tumor tissues revealed that DMF enhanced the infiltration and cytotoxic function of TILs (Fig. S7G and Fig. 7G-I). Collectively, these findings suggest that combining DMF with PD-1 blockade or TIL therapy may offer a more effective treatment strategy by enhancing CD8⁺ T cell infiltration and function, thereby improving the clinical outcomes of human CC.

## Discussion

Developing effective immunotherapies is of paramount importance for treating CC, a common gynecological malignancy with high incidence and mortality rates [4]. The findings of this study provide a novel solution by repurposing DMF, an FDA-approved drug previously used in treating autoimmune diseases, for the immunotherapy of CC. Through activating the cGAS-STING-TBK1 pathway in tumor cells, DMF enhances CD8⁺ T cell infiltration and modulates the tumor microenvironment, leading to CC regression. Considering the poor prognosis of patients with advanced CC and the limited efficacy of current immunotherapies, incorporating DMF into the existing treatment regimens may represent a promising strategy.

The direct anticancer activity of DMF has been documented dating back to 2006 in melanoma [38], and was later extended to breast cancer [21], colon cancer [39], and lung cancer [22]. In this regard, multiple mechanisms have been suggested, including the inhibition of NF-κB signaling pathway, blocking tumor cell proliferation cycles, inducing apoptosis, and suppressing the expression of matrix metalloproteinase [18, 21, 22, 40]. In CC, DMF has also been found to be capable of inhibiting the proliferation of human HeLa cells in vitro [40]. In line with this, we did observe the direct tumor-suppressing activity of DMF toward murine CC cell line TC-1 in vitro. Although DMF at a low concentration (50 μM) demonstrated a modest in vitro tumor growth inhibition in TC-1 cells (Fig. S1A), tumor growth was not affected in tumor-bearing immunodeficient mice after low-dose DMF treatment (Fig. 1A–C). Interestingly, however, the same DMF treatment regimen resulted in significant growth inhibition of implanted tumor in immunocompetent mice (Fig. 1E–-F), suggesting that in addition to the direct anticancer activity, DMF can also indirectly result in tumor regression under the help of immune system, especially CD8^+^ T cells, as illustrated by the reverse of DMF-mediated tumor suppression upon CD8^+^ T cell-depletion (Fig. 1M). Therefore, the novel indirect anticancer activity of DMF uncovered by this study opens up a new horizon for the application of DMF in cancer therapy.

It is important to note that DMF was originally used as an anti-inflammation drug for treating patients with autoimmune diseases such as multiple sclerosis and psoriasis [14, 41], therefore might raise concerns about its systemic application in cancer patients, which could potentially compromise antitumor immune response. Indeed, DMF has been reported to inhibit autoreactive T helper (Th) 1 and Th17 cells in autoimmune disorders in both mouse models and human [42, 43]. However, several lines of evidence provide by us may help to relieve these concerns. First, in tumor-free mice, DMF treatment did not cause changes in the proportion of major immune cells including those generally considered as immunosuppressive. Second, in our tumor model, increased frequencies of both IFN-γ⁺CD4⁺ T (Th1) cells (Fig. 1J) and IL-17A⁺CD4⁺ T (Th17) cells (Fig. S1G) were observed after DMF treatment, which are in contrast to those noted in autoimmune disease. Thus, these results suggest that the immunomodulatory effects of DMF may be disease-specific and context-dependent. The tumor microenvironment is characterized by persistent immunosuppression and chronic antigen stimulation, conditions that may alter the balance of T cell differentiation such that DMF, while relieving immunosuppression, also promotes the recruitment, activation and differentiation of a variety of effector T cells (Th1, Th17, and CTL). It’s important to note that while Th1 cells and CTLs are generally considered as anti-tumoral, the role of Th17 cells in cancers is complex, as they can enhance anti-tumor immunity through pro-inflammatory cytokines but may also promote tumor progression under certain conditions [44, 45]. Therefore, the net impact of Th17 upregulation in response to DMF treatment warrants further investigations.

The direct or indirect effect of DMF on CD8^+^ T cells has not been reported in cancers, and even less-explored in the context of autoimmune diseases. A study by Lückel et al. found that, in a murine EAE model, DMF treatment reduced the proportion of proinflammatory Tc17 cells and drove CD8⁺ T-cell differentiation toward a CTL phenotype with increased cytotoxic function [46]. In concordance with this observation, we found in the current study that naïve CD8^+^ T cells simultaneously exposed to activation stimuli (anti-CD3/CD28/IL-2) and DMF exhibited enhanced cytotoxicity but concurrently decreased proliferation compared to those exposed to activation stimuli alone (Fig. S3B-D). Although the differentiation states of the CD8⁺ T cells subjected to DMF in these two studies are different (Tc17 vs naïve), taken together these results suggest that CD8⁺ T cells respond to DMF differently from Th cells, which show not only inhibited proliferation but also suppressed Th1 differentiation after DMF treatment [47, 48]. Indeed, the difference in metabolic requirements lies between CD8^+^ and CD4^+^ T cells [49], and DMF are known to be capable of regulating metabolism in immune cells [50, 51]. However, further experiments are required to validate the above speculation.

Nevertheless, both increased infiltration and enhanced cytotoxicity of CD8^+^ T cells in CC tissue were observed in tumor-bearing mice after DMF treatment in this study (Fig. 1I-K). Although the direct cytotoxicity-promoting effect of DMF on CD8^+^ T cells cannot be excluded in this scenario as previously discussed, several lines of evidence suggests that these alterations are largely attributable to the indirect effect of DMF on CC cells. First, although in vitro treatment of CD8^+^ T cells with DMF did increase their cytotoxic differentiation, it also inhibited their proliferation, leading to the overall decreased CTL numbers, which is different from the observation in vivo. Second, when DMF-treated and -untreated TC-1 cells were implanted into immunocompetent mice, an experimental system in which CD8^+^ T cells were not exposed to DMF, similar alterations CD8^+^ T cells were only observed in the DMF-treated but not -untreated group (Fig. 2G–L). Finally, elimination of mtDNA, STING, and CCL5/CXCL10 in DMF-treated TC-1 cells counteracted these effects. Therefore, these evidences, together with the well-known activity of cGAS-STING-IFN I pathway in promoting CTL differentiation, lend a strong support for the hypothesis that DMF promotes CD8^+^ T cell-mediated antitumor response and thereby tumor regression through activating mtDNA-cGAS-STING-IFN I-CCL5/CXCL10 pathway in tumor cells.

Interestingly, we also observed a concurrent increase in CD8⁺ T cells and monocytic myeloid-derived suppressor cells (M-MDSCs) after DMF treatment. Although this co-increase may seem paradoxical, it likely reflects the dynamic balance between immune activation and suppression in tumors. MDSCs can directly inhibit T cell cytotoxicity by perturbing T cell receptor signaling and metabolic programs, and indirectly restrain CD8⁺ T cells by promoting Treg differentiation via factors such as TGF-β and IL-10 [52]. Their expansion can be driven by tumor-derived cytokines that promote myelopoiesis and block maturation, or by mediators from activated T cells and stromal cells that stimulate MDSC activation [53]. The rise in M-MDSCs observed here may therefore represent a negative feedback response to heightened CD8⁺ T-cell cytotoxicity. Nevertheless, the pronounced tumor regression in DMF-treated mice indicates that the CD8⁺ T cell-mediated antitumor immunity ultimately outweighed MDSC mediated suppression in the context of DMF treatment, shifting the balance toward tumor control.

Although the pivotal role of cGAS-STING signaling pathway in inducing innate immune response has been firmly established [54, 55], its effect on the outcomes of inflammation-related diseases including cancer could be fundamentally distinct and even opposite. Recently, much effort has been put in reconciling the distinct impact of cGAS-STING activation on cancers by hypothesizing that while transient engagement of cGAS-STING results in acute inflammation and thereby leads to tumor control, sustained activation of this pathway and the consequent chronic inflammation may instead promote tumor progression [56, 57]. In this context, multiple factor such as the cell types with cGAS-STING activation (e.g., tumor cells, accessory cells, and T cells), the activation/differentiation status of these cells, the source of STING agonists (e.g., mtDNA, nucDNA, and artificial chemical compounds), and cancer stages (precancerous, early and late) might affect the inflammation status and hence distinct disease outcomes. For example, in renal epithelial cells, mitochondrial fumarate accumulation caused by fumarate hydratase (FH) deficiency can lead to constant leakage of mtDNA into the cytoplasm, thereby activating the cGAS-STING-TBK1 signaling axis and triggering a continuous innate immune response. This mechanism has been proven to be closely related to the pathogenesis of hereditary leiomyomatosis and renal cell carcinoma (HLRCC) [58]. In contrast, we found that DMF treatment is effective in treating CC through activating cGAS-STING-TBK1 pathway in mouse tumor models. Several explanations can be offered to reconcile this seemly discrepancy. First, cancer type and stages are different in these two studies (developed CC vs developing HLRCC); Second, DMF treatment of CC induces a transient activation of mtDNA-cGAS-STING pathway, while FH deficiency in renal epithelial cells results in constantly elevated mtDNA-cGAS-STING response, likely leading to acute and chronic inflammation, respectively. Therefore, transiently activating cGAS-STING pathway by DMF treatment may represent a promising therapeutic drug for treating CC. Indeed, high expression of *STING* is positively correlated with the prognosis of human CC (Fig. S7D). Furthermore, as an FDA-approved drug with documented safety profiles, DMF functions through inducing the leakage of endogenous mtDNA into cytoplasm, distinguishing it from current exogenous STING agonists that are mostly artificial chemical compounds with safety profiles remained to be examined, thus making this therapeutic strategy even more attractive. However, given the aforementioned complexity regarding the role of cGAS-STING in cancer, cautions should be taken before translating it into clinical practice and extending it to other cancers.

It’s important to note that while in our study, DMF primarily triggers a transient activation of the cGAS–STING–TBK1 axis through inducing mtDNA release to promote anti-tumor immunity, a study by Xiong et al. reported a STING pathway-suppressing role of DMF in a non-tumor, acute sterile inflammatory settings [59]. This discrepancy is not necessarily contradictory: In the study by Xiong et al., mtDNA and other sources of DNA were introduced into cytoplasm before DMF treatment to mimic a pre-existing STING-induced sterile inflammation, and the target of DMF is STING and its downstream pathway; whereas in our experimental system, mtDNA exclusively resides in mitochondria of cancer cells before DMF treatment, and is triggered to be released into cytoplasm upon DMF application. Therefore, these findings suggest the multifaceted effect of DMF on diverse modules of inflammatory pathway during different stages of inflammation, which may lead to distinct outcomes of inflammation (activation or resolution). However, further direct comparative studies are required to dissect the complex role of DMF in inflammation.

The mechanism by which DMF promotes tumor regression resembles the adjuvant effect of chemotherapy or radiotherapy [60]. Since combined strategies involving chemoradiotherapy and immunotherapy are currently undergoing clinical trials for various cancer types [61–64], the low side effect profile of DMF thus make it a strong candidate for combinatorial therapies with current immunotherapies. Indeed, our findings demonstrate that DMF enhances the efficacy of PD-1 blockade and adoptive TIL therapy in mouse models. These synergic effects could be attributable to the following mechanisms. On the one hand, DMF treatment may increase the infiltration and cytotoxic differentiation of either PD-1 blockade-reinvigorated CTLs or adoptively transferred TILs, thus make both immunotherapies more effective. On the other hand, STING activation might upregulate PD-L1 expression in tumor cells [65], which could in turn increase tumors’ sensitivity to ICB therapy. Given the limited efficacy of ICB monotherapy in CC (with response rates of only 14.6%-27.8%) [6], and the diverse patient response to TIL therapy (ranging from no-responsive to complete tumor regression), the synergistic effect of DMF with ICB and TILs demonstrated in this study provides insight into developing novel effective combinatorial strategies in treating advanced CC.

Despite these promising prospects, several challenges remain. The effects of tumor heterogeneity, immune evasion mechanisms, and the potential systemic inflammation caused by prolonged STING activation on DMF treatment need to be carefully examined in further investigations. Moreover, future clinical trials are warranted to optimize DMF dosing regimens and examine the therapeutic efficacies of DMF monotherapy and its combination with TIL therapy or immune checkpoint inhibitors.

## Conclusions

Dysregulation of immune cell chemotaxis in solid tumors remains a central problem to effective cancer immunotherapy. Our study discovered that DMF is capable of reprogramming CC cells to promote CD8^+^ T cell-dependent anti-tumor immunity through activating the mtDNA-cGAS-STING pathway. DMF treatment induces mitochondrial dysfunction in CC cells, leading to the release of mtDNA into cytoplasm, which activates the cGAS-STING pathway and type I interferon response, and subsequently induces the secretion of CCL5 and CXCL10, thereby promoting CD8^+^ T cell recruitment and cytotoxic differentiation. Importantly, in syngeneic mouse models, DMF demonstrates synergistic activity with PD-1 blockade, while in PDX models it significantly enhances TIL functionality. These findings provide viable strategies to improve the therapeutic efficacy of current immunotherapies.

## Supplementary Information

Supplementary file 1.

Supplementary file 2.

## Data Availability

The raw RNA-seq data generated for TC-1 cells in this study have been deposited in https://www.scidb.cn/ (10.57760/sciencedb.22979).

## Abbreviations

CC: Cervical cancer
DMF: Dimethyl fumarate
MMF: Monomethyl fumarate
TIL: Tumor-infiltrating lymphocyte
RNA-seq: RNA sequencing
mtDNA: Mitochondrial DNA
PDX: Patient-derived xenografts
ICBs: Immune checkpoint blockades
TCR-T: T cell receptor-gene engineered T cell
NCG: NOD/ShiLtJGpt-Prkdcem26Cd52Il2rgem26Cd22/Gpt
NAC: N-acetylcysteine
DMSO: Dimethyl sulfoxide
WT: Wild-type
GSEA: Gene set enrichment analysis
GO: Gene ontology
MACS: Magnetic-activated cell separation
HIS-SIM: High Intelligent and Sensitive SIM
FBS: Fetal bovine serum
PDXO: PDX-derived organoid
KO: Knockout
nucDNA: Nuclear DNA
EthBr: Ethidium bromide
DDC: 2′-3′-Dideoxycytidine
TC-1ρ0: MtDNA-depleted TC-1 cells
SiHa^DDC^: MtDNA-depleted SiHa cells
Th: T helper
EAE: Experimental autoimmune encephalomyelitis
CTL: Cytotoxic T lymphocyte
FH: Fumarate hydratase
HLRCC: Hereditary leiomyomatosis and renal cell carcinoma
