# Supplementary material for: Dimethyl fumarate reprograms cervical cancer cells to enhance antitumor immunity by activating mtDNA-cGAS-STING pathway
Source: J Biomed Sci. 2025 Oct 20;32:92. doi: 10.1186/s12929-025-01187-x (PMC12538808; doi:10.1186/s12929-025-01187-x)
Supplement: Supplementary file 1 [file 12929_2025_1187_MOESM1_ESM.docx]

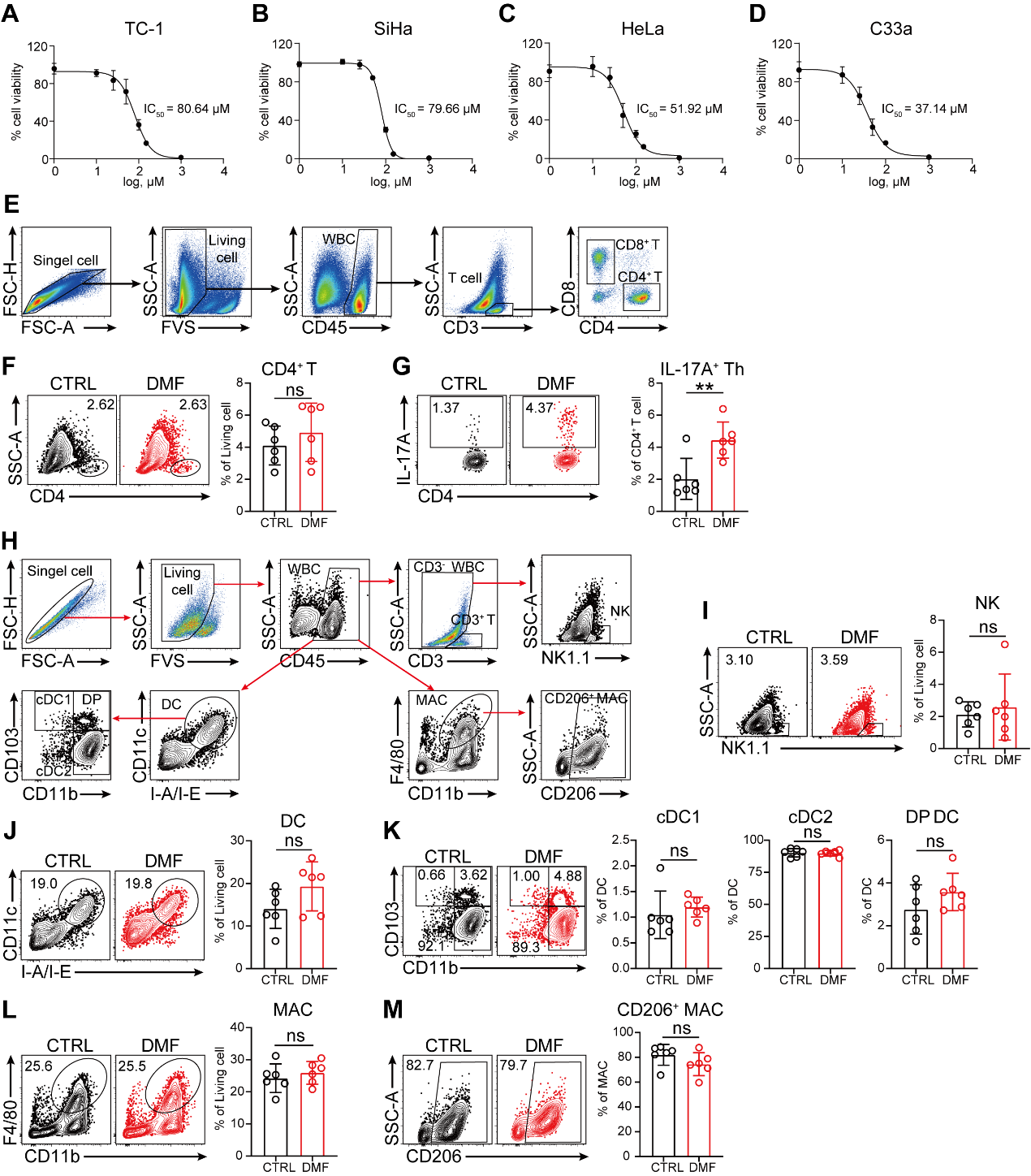


**Fig S1.** **A-D**, Cell viability curves of cervical cancer cell lines TC-1 (**A**), SiHa (**B**), HeLa (**C**), and C33a (**D**) treated with different concentrations of DMF for 24 h. **E**, Flow cytometry analysis workflow for tumor tissues from mice. **F**, Percentage of infiltrating CD4⁺ T cells in tumor of immunocompetent mice. **G**, Percentage of infiltrating IL-17A^+^ T cells in tumor of immunocompetent mice. (n = 6). **H-M**, Flow cytometry was performed to assess immune cell subsets within the tumor tissue after DMF treatment for 10 days. **H,** Flow cytometry gating strategy. **I-M,** Representative flow cytometry plots (left) and quantification of proportions (right) for NK cells (**I**), CD11c⁺MHCII⁺ DCs (**J**), CD103⁺CD11b⁻ cDC1, CD103⁻CD11b⁺ cDC2, CD103⁺CD11b⁺ double-positive DC (DP DC) (**K**) and CD11b⁺F4/80⁺ macrophages (**L**), CD206⁺ macrophages (**M**). (n = 6). Data are the mean ± SD. *P* values were determined by unpaired t-test (**F, G, I-M**); ***P* < 0.01, and ns indicating no significant difference.


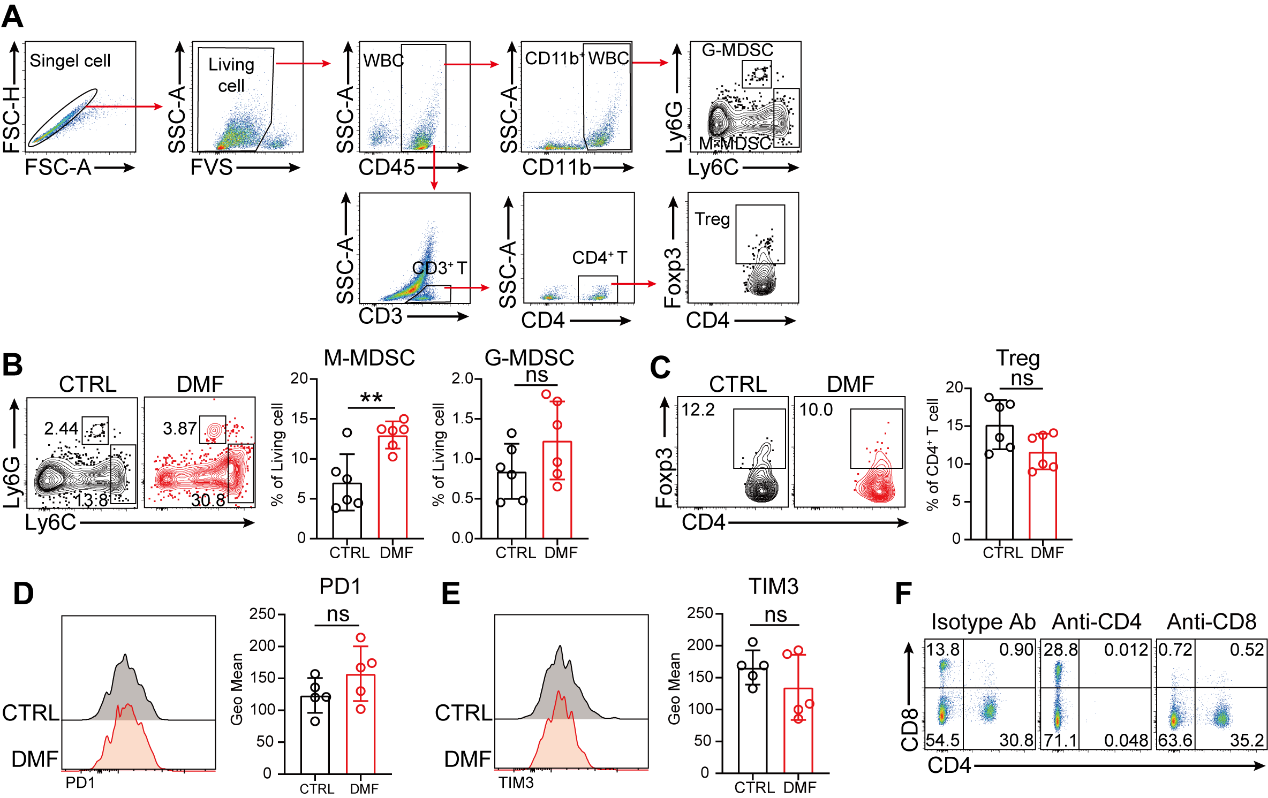


**Fig S2.** **A-C**, Flow cytometry was performed to assess immunosuppressive cell subsets within the tumor tissue after DMF treatment for 10 days. **A**, Flow cytometry gating strategy. **B-C**, Representative flow cytometry plots (left) and quantification of proportions (right) for G-MDSCs and M-MDSCs (**B**), as well as Tregs (**C**). (n = 6). **D** and **E**, Flow cytometry analysis of PD1 (**D**) and TIM3 (**E**) expression on CD45⁻ cells in tumor tissue. (n = 5). **F**, Proportion of CD4⁺ or CD8⁺ T cells in the spleens of mice after treatment with CD4/CD8 depleting antibodies. Data are presented as the mean ± SD. *P* values were calculated using unpaired two-tailed Student’s t test (**B**-**E**), ***P* < 0.01, and ns indicating no significant difference.


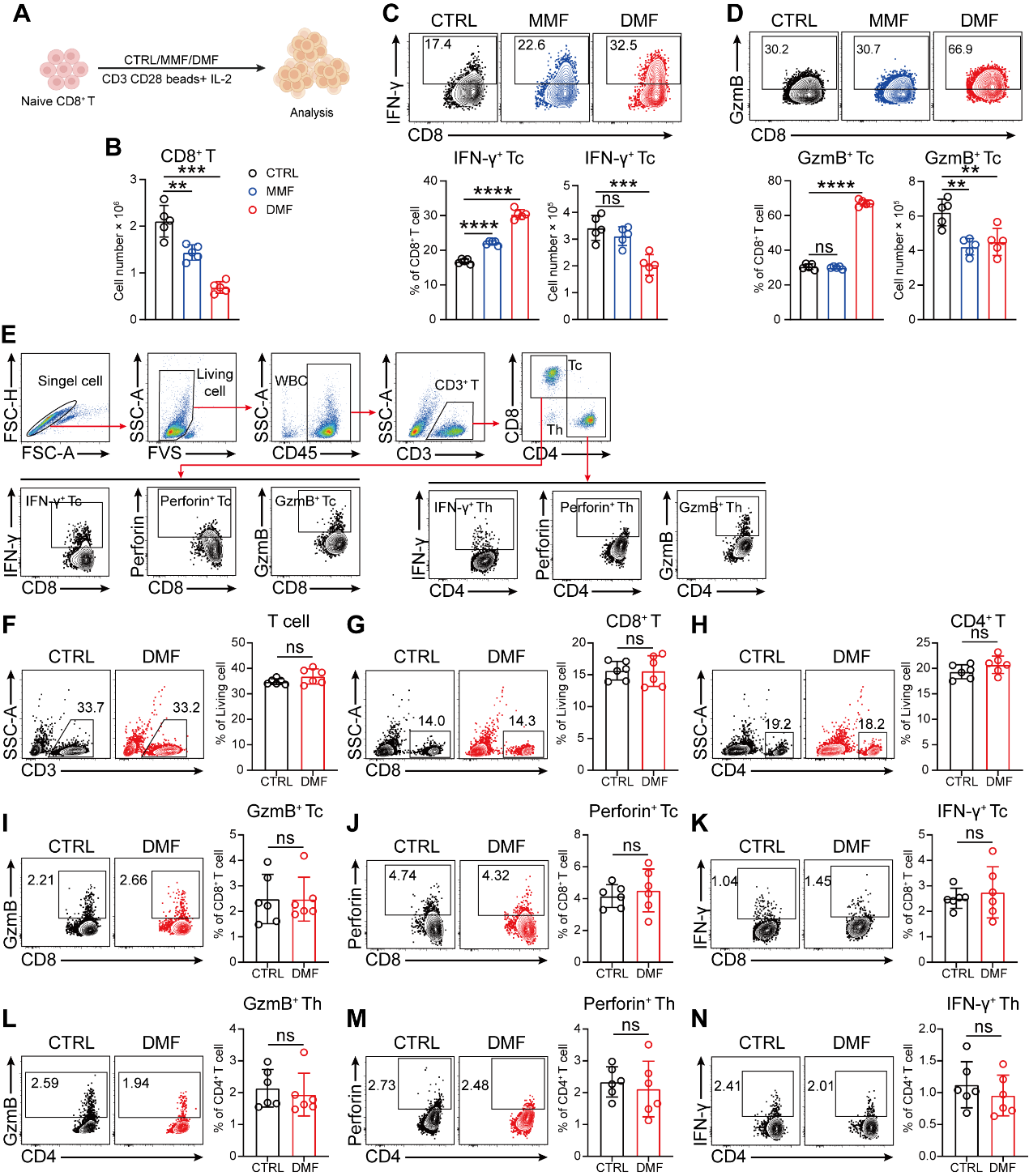


**Fig S3.** **A**-**D**, Naïve CD8^+^ T cells were treated with 100 μM MMF or 50 μM DMF, and simultaneously cultured with CD3&CD28 beads and IL-2 in lymphocyte culture medium for 72 h (**A**). The number of T cell (**B**), the number and percentage of IFN-γ^+^ CD8^+^ T cell (**C**), and GzmB^+^ CD8^+^ T cell (**D**) were measured. **E-N**, C57BL/6 immunocompetent mice without tumor inoculation were orally administered DMF or vehicle control for 10 consecutive days. Flow cytometry was performed to assess differences in peripheral blood T cells and their subsets: **E**, flow cytometry gating strategy; **F-N**, representative flow cytometry plots (left) and quantification of proportions (right) for total T cells (**F**), CD8⁺ T cells (**G**), CD4⁺ T cells (**H**), GzmB⁺CD8⁺ T cells (**I**), Perforin⁺CD8⁺ T cells (**J**), IFN-γ⁺CD8⁺ T cells (**K**), GzmB⁺CD4⁺ T cells (**L**), Perforin⁺CD4⁺ T cells (**M**), and IFN-γ⁺CD4⁺ T cells (**N**). (n = 6). Data are the mean ± SD. *P* values were calculated using unpaired two-tailed Student’s *t* test (**F-N**), one-way ANOVA for Dunnett’s multiple comparisons test (**B**-**D**). ***P* < 0.01, ****P* < 0.001, *****P* < 0.0001, and ns indicating no significant difference.


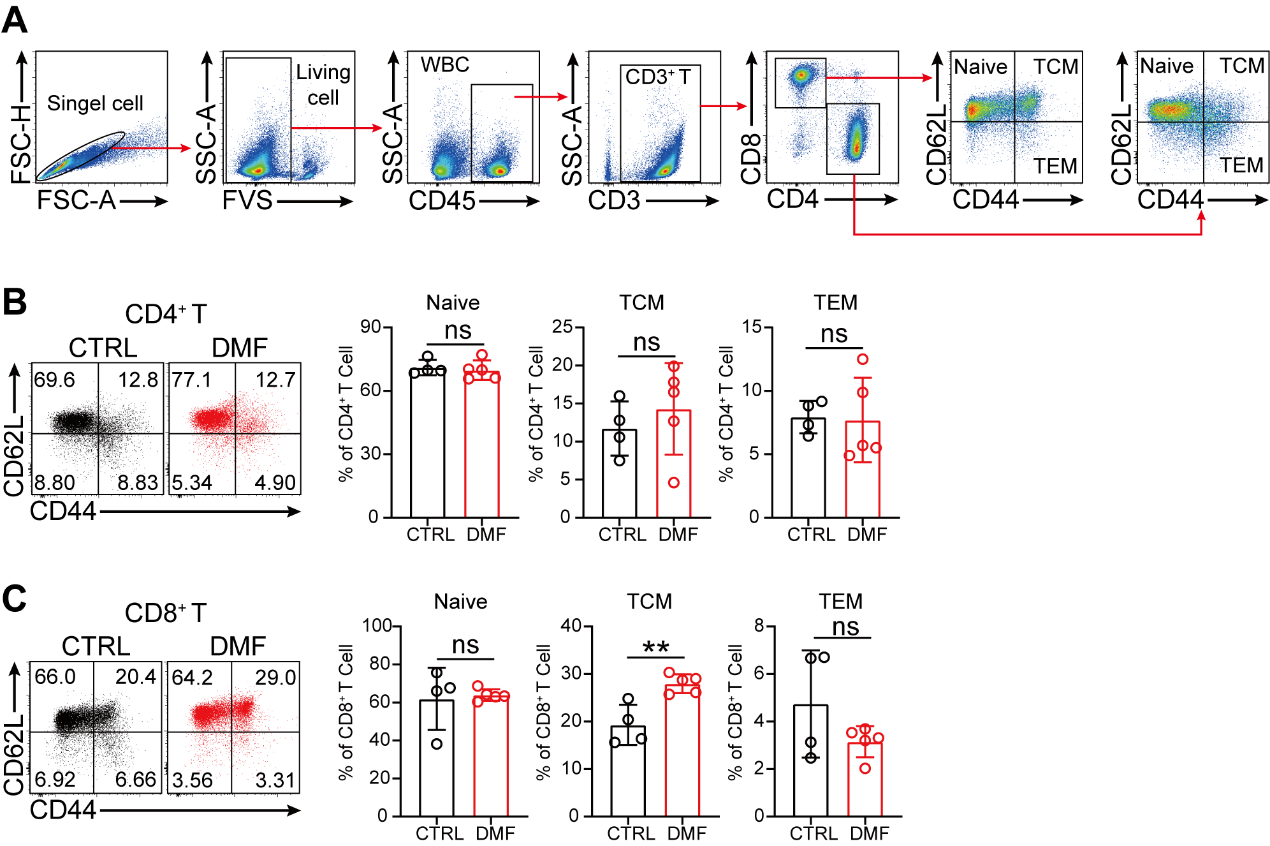


**Fig S4.** **A**-**C**, TC-1 tumor cells were treated in vitro with 50 μM DMF or an equivalent dose of DMSO for 24 h. Tumor cells from each treatment group were then subcutaneously inoculated into immunocompetent mice. 14 days later, flow cytometry was performed to assess the proportions of T cell memory subsets in tumor-draining lymph nodes: **A,** flow cytometry gating strategy; **B-C,** representative flow cytometry plots (left) and quantification of proportions (right) for CD4⁺ T cell memory subsets (**B**) and CD8⁺ T cell memory subsets (**C**). (n = 5). *P* values were determined by unpaired t-test; ***P* < 0.01, and ns indicating no significant difference.


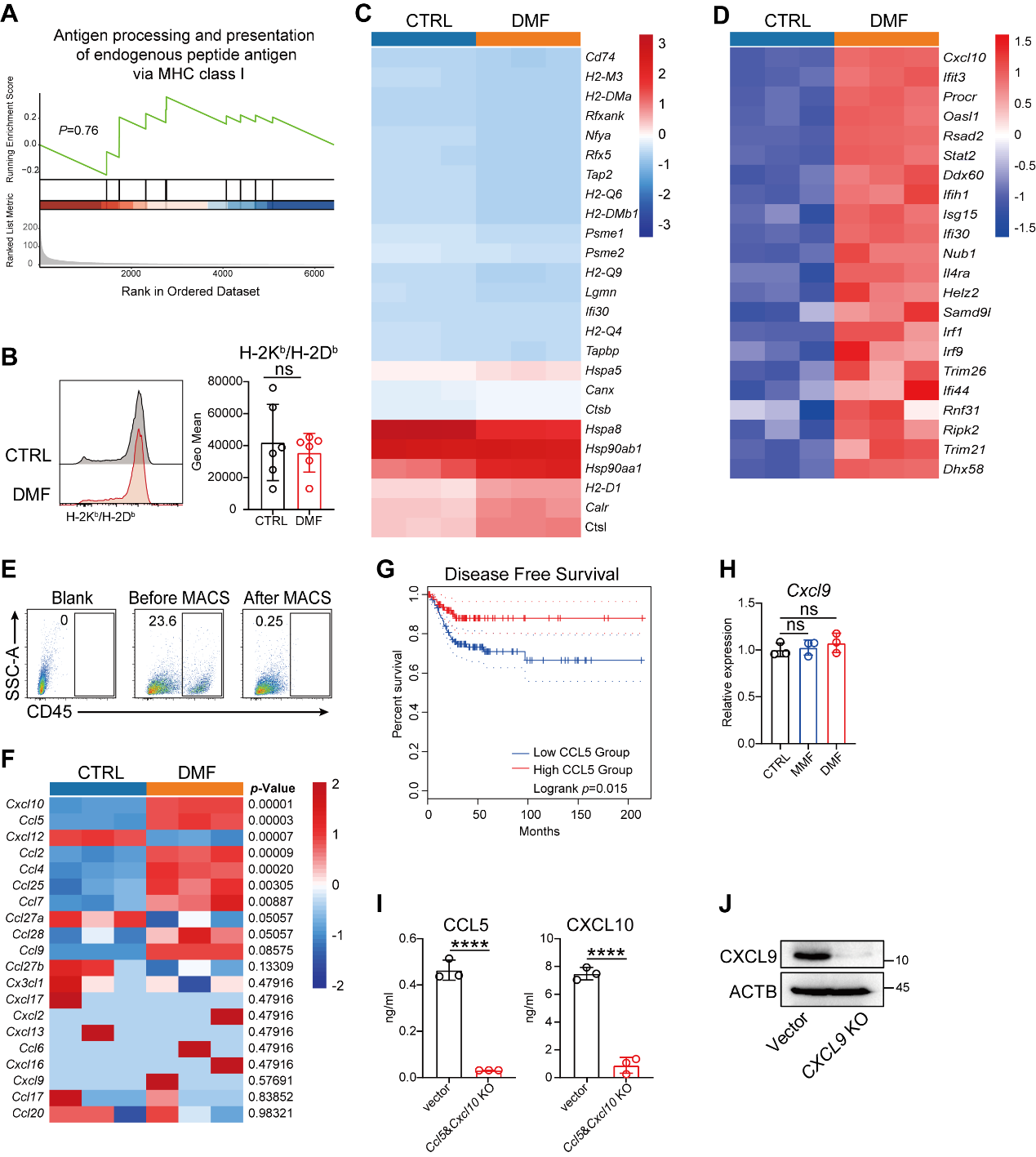


**Fig S5.** **A**, Enrichment analysis of differentially expressed genes in the MHC class I antigen processing and presentation pathway (GO:0019885) after treatment of TC-1 cells with 50 μM DMF for 24 h. **B**, Flow cytometry analysis of MHC I expression on the surface of CD45⁻ cells in tumor tissue (n = 6). **C**, Heatmap of genes related to MHC class I antigen presentation in TC-1 cells treated with 50 μM DMF for 24 h. **D**, Heatmap of differentially expressed genes related to the type I interferon signaling pathway. **E**, Flow cytometry analysis of the purity of CD45⁻ tumor cells in tumor tissue before and after magnetic bead sorting. **F**, Chemokine gene expression in TC-1 cells treated with or without 50 μM DMF. **G**, Kaplan–Meier analysis comparing disease-free survival in cervical cancer patients with low versus high *CCL5* gene expression, analyzed using GEPIA2 (http://gepia2.cancer-pku.cn/). **H**, Transcriptional levels of *Cxcl9* in TC-1 cells treated with or without 100 μM MMF or 50 μM DMF for 24 h, analyzed by qRT-PCR. **I**, Concentrations of CXCL10 and CCL5 in the supernatant of TC-1 vector control or *Ccl5*&*Cxcl10* KO cells. **J**, Western blot analysis of Cxcl9 protein levels in vector control and *Cxcl9* knockout TC-1 cells. Data are the mean ± SD. *P* values were calculated using unpaired two-tailed Student’s *t* test (**B** and **I**), one-way ANOVA for Dunnett’s multiple comparisons test (**H**). *****P* < 0.0001, and ns indicating no significant difference.


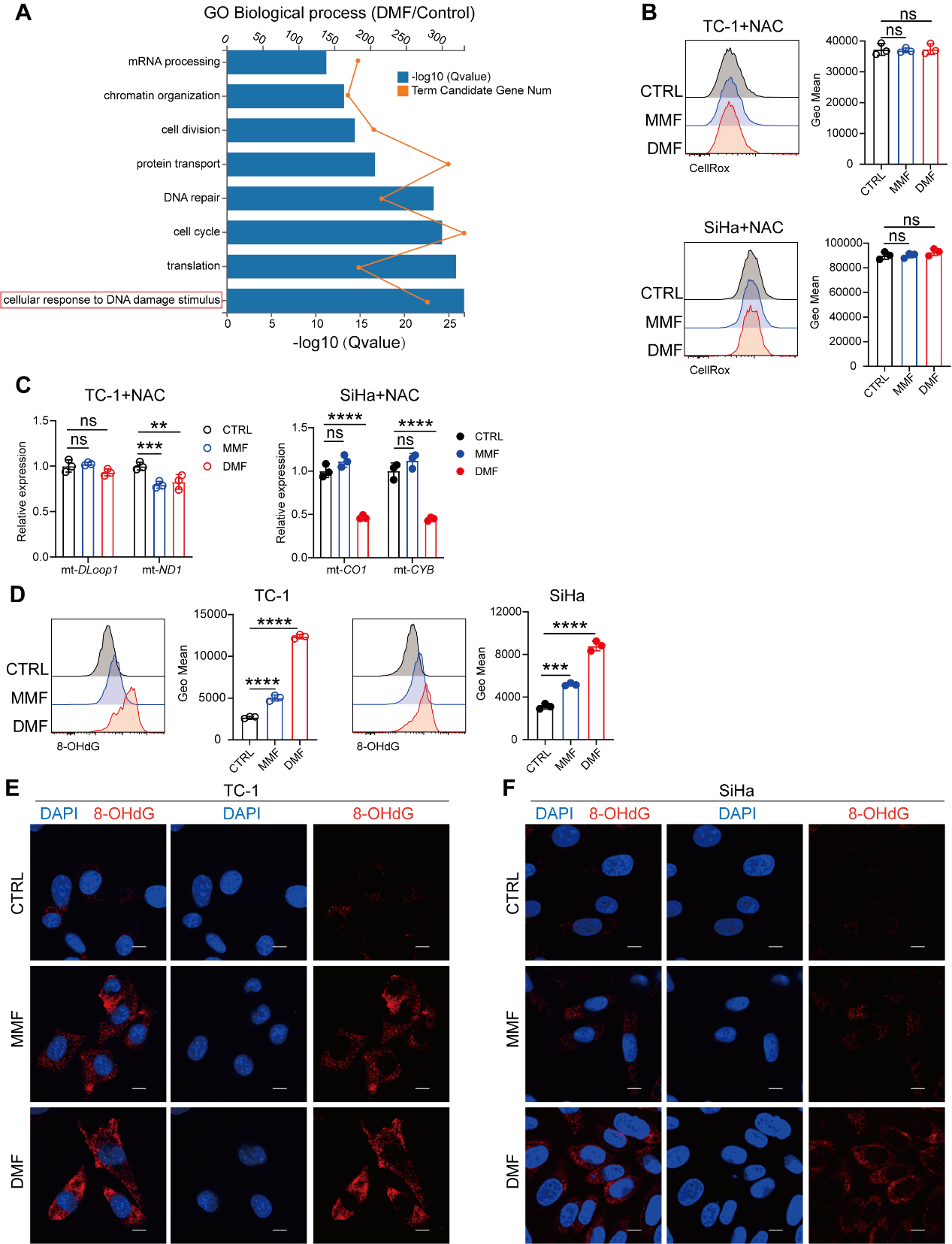


**Fig S6.** **A**, GO enrichment analysis of TC-1 cells treated with or without 50 μM DMF. **B** and **C**, TC-1 or SiHa cells were exposed to 10 mM NAC for 5 h, followed by the addition of 100 mM MMF or 500 mM DMF. **B**, CellROX levels were measured 12 h after drug treatment. **C**, Relative expression levels of mtDNA outside the mitochondria in the cytoplasm were detected by qRT-PCR 24 h after drug treatment. **D**, Flow cytometric analysis of intracellular 8-OHdG levels in tumor cells following treatment with 50 mM MMF or 100 mM DMF for 24 h. **E**-**F**, Immunofluorescence image of tumor cells treated with 100 μM MMF or 50 μM DMF for 24 h, (**E**) showing TC-1 cells, (**F**) showing SiHa cells. 8-OHdG, red; DAPI, blue; scale bar = 10 μm. Data are the mean ± SD. *P* values were calculated using one-way ANOVA for Dunnett’s multiple comparisons test (**B** and **D**), two-way ANOVA for Tukey’s multiple comparisons test **C**). ****P* < 0.001, *****P* < 0.0001, and ns indicating no significant difference.


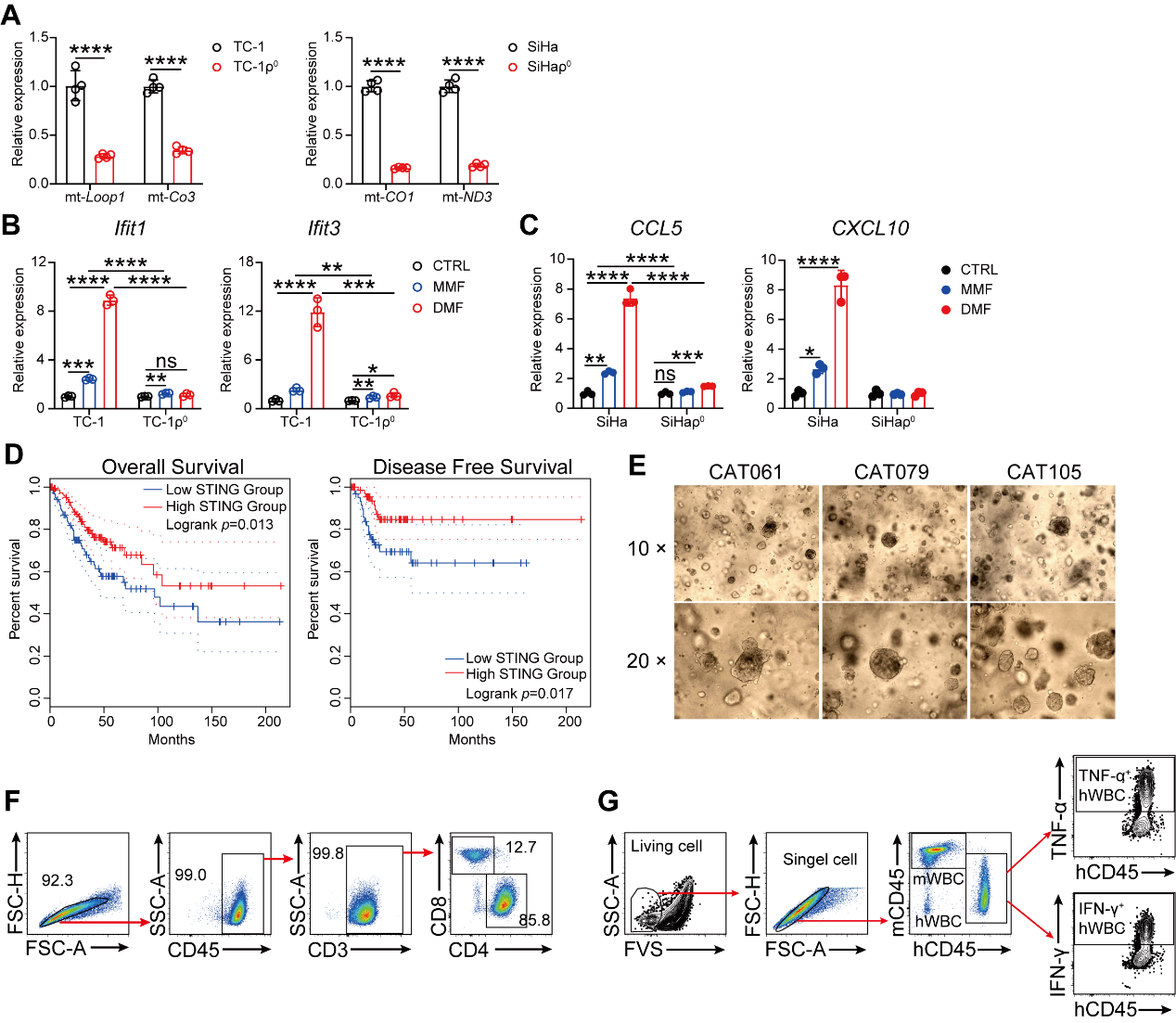


**Fig S7. A**, mtDNA content was assessed by qRT-PCR following 20 days of treatment with 100 ng/ml EthBr in TC-1 cells or 100 nM DDC in SiHa cells. **B**, Transcriptional levels of *Ifit1* and *Ifit3* were quantified by qRT-PCR in TC-1 and TC-1ρ^0^ cells treated with 100 μM MMF or 50 μM DMF for 24 h. **C**, Transcriptional levels of *CCL5* and *CXCL10* were quantified by qRT-PCR in SiHa and SiHaρ^0^ cells treated with 100 μM MMF or 50 μM DMF for 24 h. **D**, Kaplan-Meier analysis comparing overall survival and disease-free survival in CC patients with low versus high *STING* gene expression. Analysed with <http://gepia2.cancer-pku.cn/>. **E**, Representative images of organoids derived from cervical cancer patients, captured under 10 × and 20 × magnification. (n = 3 patients). **F**, TIL subpopulations expanded ex vivo from cervical cancer patient was analyzed by flow cytometry. Data are presented as the mean ± SD. **G,** Flow cytometry gating strategy for PDX samples. Data are the mean ± SD. *P* values were calculated using two-way ANOVA for Tukey’s multiple comparisons test (**A**-**C**), with significance levels defined as **P* < 0.05, ***P* < 0.01, ****P* < 0.001, *****P* < 0.0001, and ns indicating no significant difference.
