# Supplementary material for: Dimethyl fumarate reprograms cervical cancer cells to enhance antitumor immunity by activating mtDNA-cGAS-STING pathway
Source: J Biomed Sci. 2025 Oct 20;32:92. doi: 10.1186/s12929-025-01187-x (PMC12538808; doi:10.1186/s12929-025-01187-x)
Supplement: Supplementary file 2 [file 12929_2025_1187_MOESM2_ESM.docx]

**Table 1. qRT-PCR primer**

| **Oligo Name** | **Sequence** |
| --- | --- |
| m-Ccl5 (F) | CCTGCTGCTTTGCCTACCTCTC |
| m-Ccl5 (R) | ACACACTTGGCGGTTCCTTCGA |
| m-Cxcl10 (F) | GTTGAGATCATTGCCACGATGAAA |
| m-Cxcl10 (R) | CTGCTGTCCATCCATCGCA |
| m-Ifit1 (F) | AGAGTCAAGGCAGGTTTCTG |
| m-Ifit1 (R) | TGTGAAGTGACATCTCAGCTG |
| m-Ifit3 (F) | TTCCCAGCAGCACAGAAAC |
| m-Ifit3 (R) | AAATTCCAGGTGAAATGGCA |
| m-Actb (F) | CATTGCTGACAGGATGCAGAAGG |
| m-Actb (R) | TGCTGGAAGGTGGACAGTGAGG |
| h-CCL5 (F) | CCTGCTGCTTTGCCTACATTGC |
| h-CCL5 (R) | ACACACTTGGCGGTTCTTTCGG |
| h-CXCL10 (F) | GGTGAGAAGAGATGTCTGAATCC |
| h-CXCL10 (R) | GTCCATCCTTGGAAGCACTGCA |
| h-ACTIN (F) | CACCATTGGCAATGAGCGGTTC |
| h-ACTIN (R) | AGGTCTTTGCGGATGTCCACGT |

**Table 2. DNA assay**

| **Oligo Name** | **Sequence** |
| --- | --- |
| m-nucTert (F) | CTAGCTCATGTGTCAAGACCCTCTT |
| m-nucTert (R) | GCCAGCACGTTTCTCTCGTT |
| m-mtDloop1 (F) | AATCTACCATCCTCCGTGAAACC |
| m-mtDloop1 (R) | TCAGTTTAGCTACCCCCAAGTTTAA |
| m-mtDloop2 (F) | CCCTTCCCCATTTGGTCT |
| m-mtDloop2 (R) | TGGTTTCACGGAGGATGG |
| m-mtND1 (F) | CTAGCAGAAACAAACCGGGC |
| m-mtND1 (R) | CCGGCTGCGTATTCTACGTT |
| m-mtCO3 (F) | CCTCGTACCAACACATGATCTAGG |
| m-mtCO3 (R) | AGTGGGACTTCTAGAGGGTTAAGTG |
| h-nucORF1 (F) | AGAACGCCACAAAGATACTCCTCG |
| h-nucORF1 (R) | CTCTCTTCTGGCTTGTAGGGTTTCTG |
| h-nucORF2 (F) | AAACTGAACAACCTGCTCCTGAATG |
| h-nucORF2 (R) | CTACACACTGCTTTGAATGCGTCC |
| h-mtCO1 (F) | TCCTACTTCTCCTATCTCTCCC |
| h-mtCO1 (R) | GTGCTCACACGATAAACCC |
| h-mtCYB (F) | ATCACCTTCCACCCTTACTAC |
| h-mtCYB (R) | AAGGACGCCTCCTAGTTTG |
| h-mtND3 (F) | ACGGCTACATAGAAAAATCCAC |
| h-mtND3 (R) | TGGTAGGGGTAAAAGGAGGG |

**Table 3. Lymphocyte culture medium**

| **Product** | **Concentration** | **Catalog** |
| --- | --- | --- |
| RPMI 1640 | / | Gibco (10379144) |
| FBS | 10 % | Gibco (A4766801) |
| GlutaMax | 1× | Gibco (35050087) |
| HEPES | 10 mM | Gibco (15630-106) |
| Penicillin/Streptomycin | 1 mg/ml | Beyotime (C0222） |
| IL-2 | 10 U/ml | Peprotech (AF-212-12) |
| β-mercaptoethanol | 50 μM | Sigma (444203) |

**Table 4. PDXO culture medium**

| **Product** | **Concentration** | **Catalog** |
| --- | --- | --- |
| Advanced DMEM/F12 | / | Gibco (12634010) |
| GlutaMax | 1× | Gibco (35050087) |
| HEPES | 10 mM | Gibco (15630-106) |
| Penicillin/Streptomycin | 1 mg/ml | Beyotime (C0222） |
| B27 | 1× | Gibco (17504044) |
| Human EGF | 10 ng/ml | Peprotech (AF-100-15) |
| Human Noggin | 100 ng/ml | Peprotech (120-10C) |
| Human FGF-7 | 25 ng/ml | Peprotech (100-19) |
| N-acetyl-L-cysteine9（NALC） | 1.25 mM | MCE (HY-B0215) |
| Nicotinamide（NCTA） | 2.5 mM | MCE (HY-B0150) |
| TGF-β I Receptor inhibitor（A83-01） | 500 nM | MCE (HY-10432) |
| ROCK inhibitor（Y-27632） | 10 μM | MCE (HY-10071) |
| Forskolin | 10 μM | MCE (HY-15371) |
| P38 MAPK inhibitor（SB 202190） | 1 μM | MCE (HY-10295) |
